# *Cbp80* is needed for the expression of piRNA components and piRNAs

**DOI:** 10.1371/journal.pone.0181743

**Published:** 2017-07-26

**Authors:** Ravish Rashpa, Paula Vazquez-Pianzola, Martino Colombo, Greco Hernandez, Dirk Beuchle, Fabienne Berger, Stephan Peischl, Rémy Bruggmann, Beat Suter

**Affiliations:** 1 Institute of Cell Biology, University of Bern, Bern, Switzerland; 2 Interfaculty Bioinformatics Unit and Swiss Institute of Bioinformatics, University of Bern, Bern, Switzerland; 3 Department of Chemistry and Biochemistry, University of Bern, Bern, Switzerland; 4 Division of Basic Research, National Institute of Cancer, Tlalpan, Mexico; Institut de Biologie Moleculaire et Cellulaire, FRANCE

## Abstract

Cap binding protein 80 (Cbp80) is the larger subunit of the nuclear cap-binding complex (nCBC), which is known to play important roles in nuclear mRNA processing, export, stability and quality control events. Reducing *Cbp80* mRNA levels in the female germline revealed that *Cbp80* is also involved in defending the germline against transposable elements. Combining such knockdown experiments with large scale sequencing of small RNAs further showed that *Cbp80* is involved in the initial biogenesis of piRNAs as well as in the secondary biogenesis pathway, the ping-pong amplification cycle. We further found that *Cbp80* knockdown not only led to the upregulation of transposons, but also to delocalization of Piwi, Aub and Ago3, key factors in the piRNA biosynthesis pathway. Furthermore, compared to controls, levels of Piwi and Aub were also reduced upon knock down of *Cbp80*. On the other hand, with the same treatment we could not detect significant changes in levels or subcellular distribution (nuage localization) of piRNA precursor transcripts. This shows that *Cbp80* plays an important role in the production and localization of the protein components of the piRNA pathway and it seems to be less important for the production and export of the piRNA precursor transcripts.

## Introduction

piRNAs act in a gene silencing mechanism that controls the expression and translocation of transposable genetic elements (TE). This activity is particularly important in the germline where it is needed for maintaining genome integrity [1] [2]. piRNAs form a group of small RNAs, ranging in size from 23 or 24 to 30–32 nucleotides. They induce RNA-mediated gene silencing by complementary pairing with target sequences. piRNAs are bound by Argonaute proteins of the PIWI clade. In *Drosophila* these are the P element-induced wimpy testis protein (Piwi), Aubergine (Aub) and Argonaute 3 (Ago3). These three genes and proteins are essential for the production of the piRNAs [1] [2]. piRNA-related processes in *Drosophila* are active in both the male and the female germline, but most piRNA studies focused on ovaries. Two different, but related, piRNA pathways operate in these organs. The primary piRNA biogenesis pathway is active in the germline and in the somatic cells of the ovary. In both cell types it follows similar routes and involves some common factors. While somatic cells only express Piwi, germ cells express all three *Drosophila* Piwi clade Argonaute proteins, and through them they additionally produce piRNAs by the so called secondary piRNA pathway or ping-pong amplification cycle that it is active only in the germline [1] [2] [3] [4].

Many genes coding for piRNA factors had been identified in screens for genes that are essential for ovarian development and female fertility (e.g. [5]). However, while the genes had been known for quite some time, they were only much later linked to the piRNA pathway [1] [2] [6]. Recent high throughput screens have revealed a number of additional candidate genes required for the piRNA pathway in the soma and in the germline [7] [8] [9]. Among these are the genes encoding the nuclear cap-binding complex (nCBC), *Cbp80* and *Cbp20* [7]. The Cbp80/Cbp20 heterodimer interacts primarily through residues in the Cbp20 subunit with the cap structure of mRNAs and most snRNAs. Cbp80 stabilizes this interaction and mediates further interactions with other proteins such as importins, the REF (RNA and export factor binding protein)/Aly protein, CTIF, a MIF4G-domain protein involved in translation, and the NMD (non-sense mediated decay of mRNAs) factor Upf1 [10]. Binding of nCBC to the cap takes place early in the transcription cycle and is a prerequisite for the binding of additional protein components of the RNP.

In this study we investigated in more detail the role of *Cbp80* in the piRNA pathway. We found that knocking down *Cbp80* in the germline altered the expression of several piRNA pathway components and it interfered with nuclear localization of the piRNA pathway component Piwi and with the nuage localization of Argonaute 3 (Ago3) and Aubergine (Aub), two other piRNA biogenesis factors. Importantly, mRNA levels from germline TEs became elevated in the ovary. Sequencing small RNAs after knocking down *Cbp80* in the germline further revealed that *Cbp80* is required for the primary production of germline piRNAs as well as for the secondary mechanism, the ping-pong amplification. *Cbp80* is therefore involved in the expression of Piwi pathway components and in both piRNA biosynthesis pathways that are active in the female germline.

## Materials and methods

### *Drosophila* strains, constructs and transgenic lines

Stocks and crosses were grown at 25°C on standard cornmeal-agar medium. Transgenic flies, *UASP-myc*::*Cbp80*, *UASP-Venus*::*Cbp80* and *UASP-Cbp80*::*Venus* were generated using the germline-specific phiC31 integrase transgenesis method [11] and the vector described previously [12]. *OregonR* and driver-only flies (without UAS transgene inserts) were used as wild-type reference stocks. *Cbp80 RNAi* lines were from the TRiP collection (BDSC stock 33648; Harvard) and from the Vienna *Drosophila* RNAi Center (BDSC v22332). RNAi lines were against *piwi* (BDSC v22235), *aub* (TRiP) (BDSC 35201), *mCherry* (BDSC 35787) and dsGFP (BDSC 9330). *shwhite* and *shRhi* RNAi lines were kindly provided by Fabio Mohn [13]. The *pCog—Gal4* line (2^nd^ chromosome insertion) was provided by Pernille Roth [14]. All other GAL4 driver lines were obtained from the Bloomington Stock Center. To generate the *UASP-myc*::*Cbp80* transgenic line, the *Cbp80* ORF was PCR-amplified from LD31211 (BDGP cDNA collection) using primers containing a Xba1 site. The fragment was inserted into the *pUASP-myc-K10-attB* vector [15]. Cloning was in frame with the *myc-*tag. *pUASP-Jupiter*::*mCherry* flies were provided by R. Koch and R. Nag [16].

To construct the C-terminal Cbp80::Venus fusion reporter, the *Venus* ORF was amplified with primers containing Xba1 sites, and *Cbp80* was amplified with a forward primer containing a BamH1 site and a reverse primer containing an Xba1 site. Both sequences were first cloned into the pCRTopo vector. *Cbp80* was cut with BamH1 and Xba1 and subcloned into the pUASp-K10 vector to generate pUASP-C-Cbp80-K10 vector. Then *Venus* was cut from the pCRTopo vector with Xba1 and cloned into the Xba1 site of pUASP-C-Cbp80-K10 to generate *pUASP-Cbp80*::*Venus-K10* (*Cbp80*::*Venus*). For the N-terminal fusion construct *Venus* was amplified with primers containing Not1 and BamH1 sites, and *Cbp80* was amplified with primers containing BamH1 and Xba1 sites. Both were initially cloned into the pCRTopo vector. *Cbp80* was then cut with BamH1 and Xba1 and subcloned into these sites in the pUASP-K10 vector to generate *pUASP-N-Cbp80-K10 vector*. Then *Venus* was cut from the pCRTopo vector with Not1 and BamH1 and cloned into these sites in the *pUASP-N-Cbp80-K10* to generate *pUASP- Venus*::*Cbp80-K10 (Venus*::*Cbp80)*.

### Immunostaining and RNA *in-situ* hybridization

Immunostaining experiments were performed as described [15] [17]. The following additional primary antibodies where used: mouse anti-Piwi P4D2 (1:100; [18]), mouse anti-Piwi P3G11 (1:500; [18]), mouse anti-Aub 4D10 (1:1,000; [19]), mouse anti-Ago3 9G3 (1:250; [20]), rabbit anti-Cbp80 (1:100; [21]), and anti-lamin ADL84 (1:500–1:300; Developmental Studies Hybridoma Bank; [22]). RNA in situ hybridization with Stellaris probes was done essentially as described in [13], but after hybridization samples were subject to immunostaining to reveal Cbp80 and Lamin proteins. Stellaris probes for detecting the 42AB sense transcripts (probe 42AB-RS labeled with CalFluor 590) and probes against the 20A transcripts (labeled with Quasar 670) were also described [13]. After washing of the probes with hybridization wash buffer, samples were washed 2 times for 5 min with SSX (2x SSC, 0.3% Triton X-100). Ovaries were then blocked with SBX (SSX plus 0.1%BSA). Incubation with primary antibodies was done in SBX at 4°C overnight, followed by 4 times 10 min washes with SSX buffer at room temperature. Ovaries were subsequently incubated for 6 h with goat anti-mouse secondary antibodies (Alexa 405; 1:200) and with goat anti-rabbit antibodies (Alexa 488; 1:400; Molecular probes; room temperature in SBX). They were then washed 4 times 10 min with SSX and mounted using Aquamount medium. Images were analyzed either with a Leica TCS-SP2, -SP5 or -SP8 confocal microscope and processed using Leica software, Photoshop and ImageJ.

### Yeast two-hybrid assays

Interactions between bait and prey proteins were detected following a yeast interaction-mating method using the strains PJ69-4a and PJ69-4alpha [23]. Diploid cells containing both bait and pray plasmids were grown on selective media (―W (Tryptophan), ―L (Leucine)) and are shown as growth control. Protein interactions were detected by replica-plating diploid cells onto selective media (―W, ―L, ―A (Alanine) or (―W, ―L, ―H (Histidine) + 30 mM 3-amino-1,2,4-triazole (3AT)). Growth was scored after 6 days of incubation at 30°C. *Piwi* (CG6122), *Cbp20* (CG12357) and *Cbp80* (CG7035), all three full length cDNAs, were subcloned into the pOAD and/or pOBD2 vectors [23]. Cloning was in-frame either with the activator domain or the DNA-binding domain sequence of *GAL4* to create the “prey” plasmids *Piwi*-AD, *Cbp20*-AD and *Cbp80*-AD, and the “bait” plasmids *Piwi*-BD and *Cbp20*-BD.

### RNA isolation from ovaries and real time qPCR

**Fig 1:**
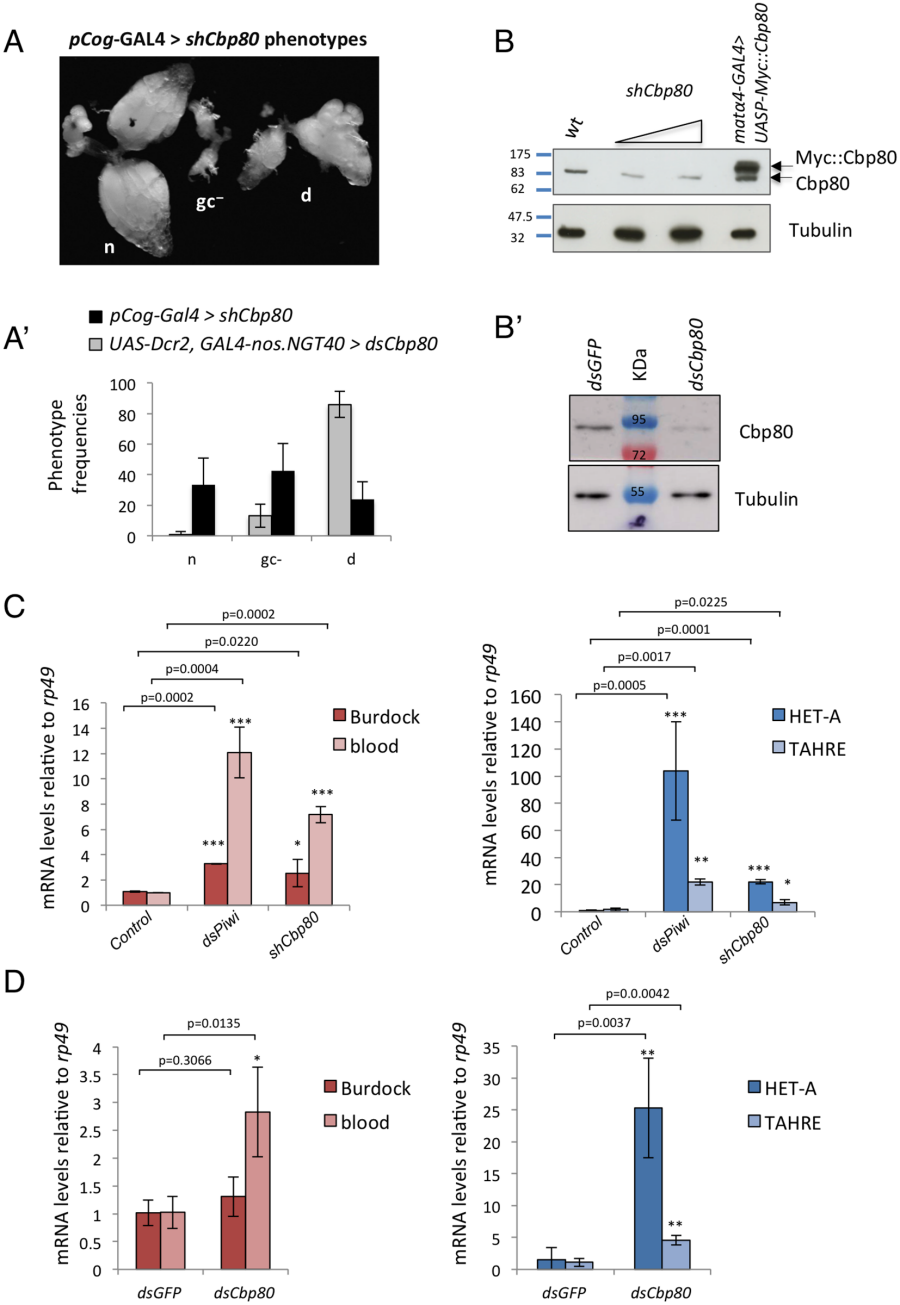
*Cbp80* phenotypes in the female germline and its role in silencing transposable elements. (**A**) Germline-specific knock down of *Cbp80* was performed either with the driver *pCog-GAL4* and an shRNA against *Cbp80 (shCbp80)* or by the GAL4-nos.NGT40 driver combined with UAS-Dcr2 and a dsRNA against *Cbp80 (dsCbp80)*. The knockdown leads to 3 phenotypic classes with the frequencies shown in (**A’**): normal wild-type phenotype [n], ovaries that appear to lack germ cells [gc^-^], and partially developing ovaries that mostly degenerate after stages 7–9 [d]. **(B-B’)** Ovaries showing the "d" phenotype upon *Cbp80* knockdown were tested for Cbp80 levels by Western blotting. The sample contained only a minor fraction of the other phentypes. In **(B)** wild-type ovaries and wild-type ovaries expressing a Myc-tagged Cbp80 were used as controls, and knock down was performed with the shRNA. 2 different amounts of the *shCbp80* samples were loaded. In **(B’)** ovaries expressing a dsRNA against *Cbp80* or *GFP* were used. Tubulin was used as a loading control. (**C**) Fold increases in RNA levels of indicated TEs upon germline-specific RNAi-mediated knock down of *piwi* and *Cbp80 (shRNA* against Cbp80). The germline GAL4 driver alone was used as control. Fold-changes in RNA levels relative to control were normalized to *rp49* levels. Error bars indicate SD; n = 3, with 2 biological replicates. (D) Fold increases in RNA levels relative to *rp49* of the same TEs upon knock down of *Cbp80* using the *dsRNA*. Control ovaries expressed the *dsGFP* RNAi construct. The upregulation of the TE was also observed when *Tub* and *BicD* were used to normalize the reads (S3 Fig). *p<0.05; **p<0.01; ***p<0.001.

Ovaries were dissected from 3–4 days old females. Total RNA from ovaries displaying the "d" phenotype (Fig 1) was extracted using TRIzol (Life technologies). cDNA for analysis of the transposon expression levels shown in Fig 1C, was prepared via oligo(dT) priming from 1μg of total RNA. For the analysis of the Piwi components and transposons levels in the remaining experiments, 100ng of total RNA, treated with the Turbo DNA-free kit, were reverse transcribed using the SuperScript III reverse transcriptase and oligo dT primers (Invitrogen). piRNA precursors transcripts were analyzed essentially as described [24]. 100 ng RNA, treated beforehand with Turbo DNA-free kit, were reverse transcribed using the SuperScript III reverse transcriptase (Invitrogen). Specific RT primers for two regions of clusters *42AB*, region A and 1–32 (plus strand primers were used) and for the *flam* locus were mixed with *rp49*-RT specific primers. All primers have been described already [24]. Real time PCR was carried out using MESA GREEN qPCR MasterMix Plus for SYBR (Eurogentec) with a Qiagen Rotor-Gene Q according to the manufacturer’s instructions. Cycle threshold (C(_T_)) values were determined by the second differential maximum method as calculated by the Rotor-Gene software. Calculation of relative mRNA levels was done by using the 2 [-ΔΔC(_T_)] method [25], where the C(_T_) values of the mRNA levels were normalized to the C(_T_) values of *rp49*, *Tub* or *BicD* mRNAs in the same sample. C(_T_) values used were the means of triplicate repeats. To test for statistical significance, we first applied a Box-Cox transformation [26]. Finally, a t-test was performed on the transformed data to obtain p-values [27]. Most primers used for RT-PCR were described previously [24] [6] [28]. Primers for *Burdock* were 5’ CGGTAAAATCGCTTCATGGT 3’ and 5’ ACGTTGCATTTCCCTGTTTC 3’. *Cbp80*, *Ago3*, *rhi* and *zuc* primers for qPCR were designed in a way that one primer spanned an intron. Primers for *Cbp80* were 5’ GGATGAGGGCTATGATCATC 3’ and 5’ TCTAGGTTCGATTCCACGGA 3’; for *rhi* 5’ ATTCCGAAGTGGAGAGCATG 3’ and 5’ CGTCATTCATCTGGTAGCAG 3’; for *Ago3* *5’* CAATTGGTACGACAGGGTAC 3’ and 5’ TGAGCGTACATACAACAAGC 3’; for *zuc* 5’ TATGCGTCCGTGCTATAGCA 3’ and 5’ CCACATTCGTTGGAATTCCG 3’; for *Aub* 5’ CGGTCATCCGGAATTTCCTCATATA 3’ and 5’ CGTGCATATCAATAGGTGGTATGTG 3’ and for *piwi* 5’ TAACGCCGAAAGATACTCATCAATC 3’ and 5’ TATCCCAACTTGCAATTCAGTTGGA 3’.

### Western analysis

Western blotting was carried out with mouse anti-Piwi P3G11 (1:1,000; [18]), mouse anti-Aub 4D10 (1:2,000; [19]), rabbit anti-Cbp80 (1:1,000; [21]), mouse anti-alpha tubulin primary antibodies (1:250; Developmental Studies Hybridoma Bank), mouse anti-BicD 1B11 (1:10; [29]), mouse anti-Cdk7 (1:10 mix of 20H5 and 19E7.2 clones; [30]) and rabbit anti-Clc (1:3000, [31]). Horseradish peroxidase-conjugated secondary antibodies were from GE Healthcare. To analyze the effects of *Cbp80* knockdown and the expression of Piwi components, ovaries with the phenotype “d” were used (Fig 1A).

### Small RNA libraries and bioinformatics analysis

Libraries were prepared with RNA extracted from ovaries. Partial *Cbp80* knockdown in the germline was achieved by pCog-Gal4 driving shRNA-*Cbp80* (TRiP line). *pCog-Gal4* driving *shmCherry* (TRiP line) in flies expressing mCherry-Jupiter in a *Jupiter*^+^ background was used as control. For the *Cbp80* knockdown we selected underdeveloped ovaries (phenotype “d”; Fig 1). RNA was extracted with TRIzol and treated with DNase I (amplification grade, Invitrogen) according to the manufacturer’s instructions. Libraries were prepared by Fasteris (Geneva, Switzerland) using the Illumina small RNA kit and a polyacrylamide size selection of 18–30 nt. The *Drosophila-*specific depletion of 2S rRNA was also performed.

Bioinformatics analyses of small RNAs were performed as described [6]. The adapter sequences were removed from the reads using Trimmomatic version 0.32 [32] without applying any further filter on base quality. Reads with lengths between 19 and 28 bp were then collapsed and aligned to the *Drosophila* genome (dm3) with Bowtie1 version 0.12.9 [33]. No mismatches and no multiple mapping were allowed. The reads per million (RPM) normalization allowed us to compare our control and experimental conditions in an unbiased way. Additionally, we also normalized knockdown conditions on the basis of the coverage of the *flamenco* locus. This normalization turned out to be very close to the RPM one, confirming that the *flamenco* locus had a very similar coverage in both the knockdown and the control. The ping-pong effect was investigated by analyzing the relative frequency (Z-score) of overlaps between reads on different strands as described in [6]. A 10 bp overlap was scored as a signature of the ping-pong effect.

## Results

### *Cbp80* is required for the biosynthesis of piRNAs

An RNAi screen for genes required for repression of transposable elements (TEs) indicated that *Cbp80* might also be required for the repression of different types of TEs [7]. Because single RNAi and high throughput screening results can sometimes be misleading due to off-target effects, we studied the function of *Cbp80* in oogenesis by targeting a different region of the *Cbp80* mRNA in the female germline. Because *Cbp80* is involved in general gene expression, it seemed likely that *Cbp80* is required for several different processes in oogenesis. To find conditions that would allow sufficient development of the germline while still showing a phenotype that resulted from reduced *Cbp80* function, we tested different *Gal4* drivers. We monitored the expression of these drivers with a *Venus*::*Cbp80* reporter in the UASP vector (S1 Fig). This method revealed that *matα4-GAL4* [34] drove expression in the vitellarial stages but not in the germarium. *pCog-GAL4* [14] on the other hand, drove expression in the germarium as well as in later stages.

To knock down *Cbp80* in the germline we then used the different *Gal4* drivers in combination with a transgenic RNAi project (TRiP) line that expresses an shRNA against *Cbp80 (shCbp80)* [35]. Using the strong but late expressing maternal tubulin driver *matα4-GAL4* resulted in ovaries with no apparent abnormalities. In contrast, *pCog-GAL4*, a driver that is expressed already in the germarium, produced ovaries that could be categorized into three distinct morphological types, normal ovaries, ovaries that lacked germ cells and ovaries that showed partial development until mid-oogenesis, but then mostly degenerating egg chambers after stages 7–9 (phenotype “d”; Fig 1A and 1A’). These phenotypes and the correlation of the phenotypes with the expression patterns of the Gal4 drivers suggest that the early expression of *Cbp80* is particularly important for the survival of the germline. Driving *Cbp80* RNAi during the germarial stages blocked germline development efficiently, whereas even the strong *matα4-GAL4* driver, which is expressed only during the vitellarial stages, did not cause any visible defects when used to knock down *Cbp80* specifically in the germline.

The same ovarian phenotypes, although with different frequencies were also observed by expressing a dsRNA that targets a different sequence of *Cbp80 (dsCbp80)* (Fig 1A’). This dsRNA and a Dicer-2 construct (*UAS-Dcr2)* were driven by the GAL4-*nos*.*NGT40* that is also expressed from the germarial stages on (S1 Fig). The fact that different lines and different RNAi constructs targeting different parts of *Cbp80* cause the same phenotypes strongly argues that the observed ovarian phenotypes are not due to off target or background effects. We interpret the phenotypic defects seen in the partially developing group (“d” in Fig 1A) as partial loss of *Cbp80* function phenotypes. Because these egg chambers still developed through the stages where specific phenotypes in early and mid-oogenesis can be studied, we focused our subsequent studies on this group. Consistent with this interpretation, these test conditions also revealed partial knockdown of Cbp80 protein by Western blotting in underdeveloped egg chambers (d), but not in the ones with wild-type appearance (n) (Fig 1B, 1B’ and S2 Fig).

To test whether germline RNA levels of transposable elements (TEs) are affected by *Cbp80*, we performed a *Cbp80* knockdown specifically in the female germline using *pCog-GAL4* to drive shRNA expression against *Cbp80*. To isolate the material for the TE expression analysis we dissected ovaries that showed the “d” phenotype (Fig 1A; see Methods section for details). Using RNA isolated from dissected wild-type and mutant ovaries we then measured levels of mRNAs of marker transposable elements. Furthermore, in order to be able to compare the *Cbp80* knockdown results with the effect of knocking down genes that are known to be required for the repression of TEs, we also knocked down *piwi* in the germline using the driver combination *matα4-GAL4; nos-GAL4*. Interestingly, like *piwi* knockdown, *Cbp80* knockdown in the germline led to de-silencing of the germline TEs blood, HET-A and TAHRE (Fig 1C and S3 Fig). However, a quantitative comparison between 2 different knockdown experiments is not possible. Confirming the role of *Cbp80* in repressing TEs, we also observed a similar upregulation of TEs using ovaries expressing the dsRNA against *Cbp80*. Furthermore, normalization to diverse mRNAs had little influence on the results (Fig 1D and S3 Fig). The combination of both results therefore provides good evidence that the *Cbp80* knockdown phenotype is not due to an off-target effect and that *Cbp80* is required for repression of TE expression in the female germline of *Drosophila*, a conclusion that is also consistent with the result observed by Czech et al. [7].

In *Drosophila*, Piwi proteins and Piwi-interacting RNAs (piRNAs) form the basis of the small RNA-mediated immunity against selfish genetic elements in the gonads [3]. To test whether *Cbp80* contributes to the piRNA pathway, we isolated and sequenced small RNAs from ovaries with reduced levels of *Cbp80* mRNA in their germline. As control, we also prepared a library from ovaries expressing a *mCherry—Jupiter* fusion construct and shRNAs against *mCherry (shmCherry)* in their germline. Both knockdowns were performed using the germline-specific driver *pCog-Gal4*. The phenotypic composition of the dissected *Cbp80* knock-down-ovaries was the same as described in the previous experiment.

**Fig 2:**
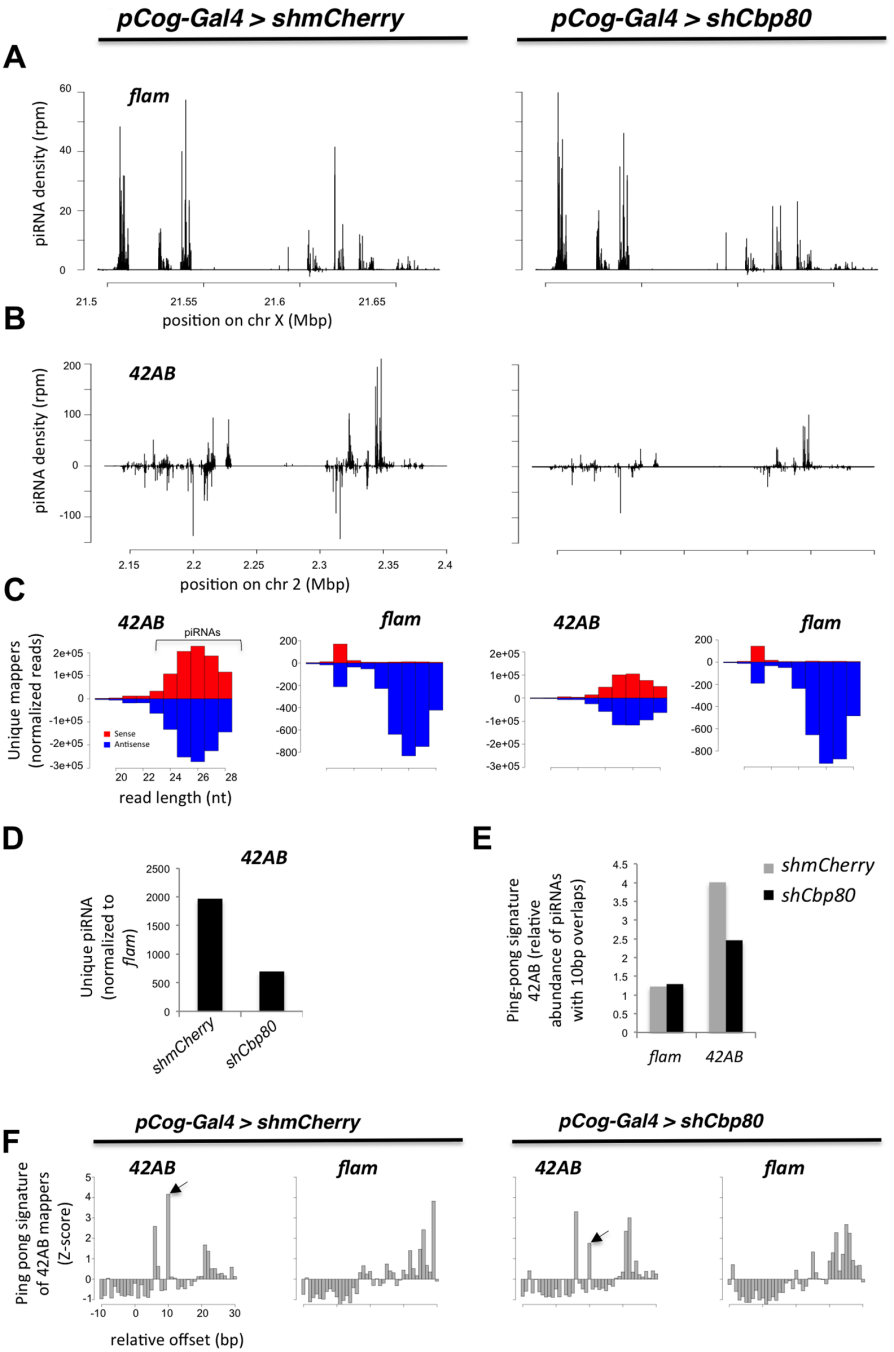
Germline *Cbp80* is involved in producing piRNAs. **(A-C)** Genotypes of ovaries analyzed are depicted on top. Ovaries showing the "d" phenotype (Fig 1A) upon *Cbp80* knockdown were used. **(A-C, F)** Scale and axis depicted in the left pannel also apply to the corresponding panels on the right. **(A-B)** Histogram showing small RNAs (23–29 nucleotides) mapping to the soma-specific *flamenco* (*flam*) cluster **(A)** and to the germline-specific cluster *42AB* **(B)**. Ovaries expressed specifically in the germline shRNAs against *Cbp80* or *mCherry* (as control). **(C)** Plots showing the size distribution of small RNAs derived from each strand of the *42AB* and the *flam* clusters. Small RNA reads derived form the *42AB* cluster were normalized to small RNAs mapping to the somatic *flam*, which is unaffected in this germline-specific knockdown. Number of small RNA reads of the characteristic size for piRNAs (23–29 nt) mapping to the germline-specific, dual-strand *42AB* cluster were reduced upon *Cbp80* knockdown. The same results were obtained when normalizing to the total number of small RNA reads. Small RNA reads derived form the *flam* cluster were normalized to the total number of small RNA reads. **(D)** A histogram showing the relative levels of *42AB* derived piRNAs upon *Cbp80* knockdown compared to the control knockdown. The data is normalized to the number of reads from the *flam* locus. Differences between control (*shmCherry*) and *shCbp80* treatment are highly significant (p-value is < 2x10^-16^ using a chi-square test). **(E)** Relative abundance of sense-antisense piRNA pairs overlapping by 10 nt (compared to the total number of sense-antisense pairs mapping to the *flam* or the *42AB* clusters, in the small RNA libraries of the *Cbp80* and the control knockdowns. Differences between *42AB* control (*shmCherry*) and *42AB* levels upon *shCbp80* treatment are highly significant (p-value is < 2x10^-16^ using a chi-square test). **(F)** Histograms showing the relative enrichment of RNAs overlapping by the indicated number of nucleotides, plotted by Z-score, for the *42AB* and *flam* clusters. Knockdown targets in the female germline are indicated on top of the figure. The peak at position 10 (arrow) is indicative of a ping-pong signature.

We analyzed the piRNA sequence reads mapping to the somatic *flamenco (flam)* locus and the ones mapping to the germline locus *42AB* (Fig 2A and 2B). Criteria for piRNA identification and bioinformatics approaches were chosen according to [6] and are described in the Methods section. Small RNA reads (of 19 to 29 bp) from the *42AB* locus were normalized to the number of reads mapping to the *flam* locus, which is unaffected by germline-specific knockdowns. Most piRNAs were in the size range from 24–28 nucleotides (nt, Fig 2C). The number of reads of this size uniquely mapping to the germline-specific, dual-strand 42AB cluster were reduced upon *Cbp80* RNAi treatment compared to the control knockdown (Fig 2C). On the other hand, small RNA reads of this size derived from the *flam* locus were unaffected as expected (Fig 2C). Levels of unique piRNAs mapping to *42AB* were reduced around 2.8 fold upon *Cbp80* knockdown (Fig 2D). This reduction was observed when data was normalized to the total number of small RNA reads or to the non-affected *flam* locus. Interestingly, *Cbp80* knockdown not only affected levels of piRNAs derived from the dual-strand *42AB* cluster, but also the piRNAs derived from the *20A* cluster, which is an uni-strand cluster (S4 Fig).

We also used the sequence data to analyze whether *Cbp80* plays a role in the ping-pong amplification cycle of the piRNA pathway. The ping-pong signature is defined as the frequency of reads from opposite strands that overlap by 10 nt. Ovaries in which genes involved in the ping-pong amplification pathway were knocked down (*spn-E*, *aub*, *del* and *shu)* showed a reduced frequency of ping-pong signatures, whereas knockdown of genes that participate mainly in the primary biogenesis pathway (*armi* and *piwi)* have no effect on it [7] [6]. Interestingly, *Cbp80* knockdown also resulted in a reduced frequency of ping-pong pairs (Fig 2E and 2F). These results therefore show that *Cbp80* plays an important role in piRNA biogenesis by affecting both the primary and the secondary piRNA biogenesis pathway.

### Expression of Cbp80 during oogenesis

**Fig 3:**
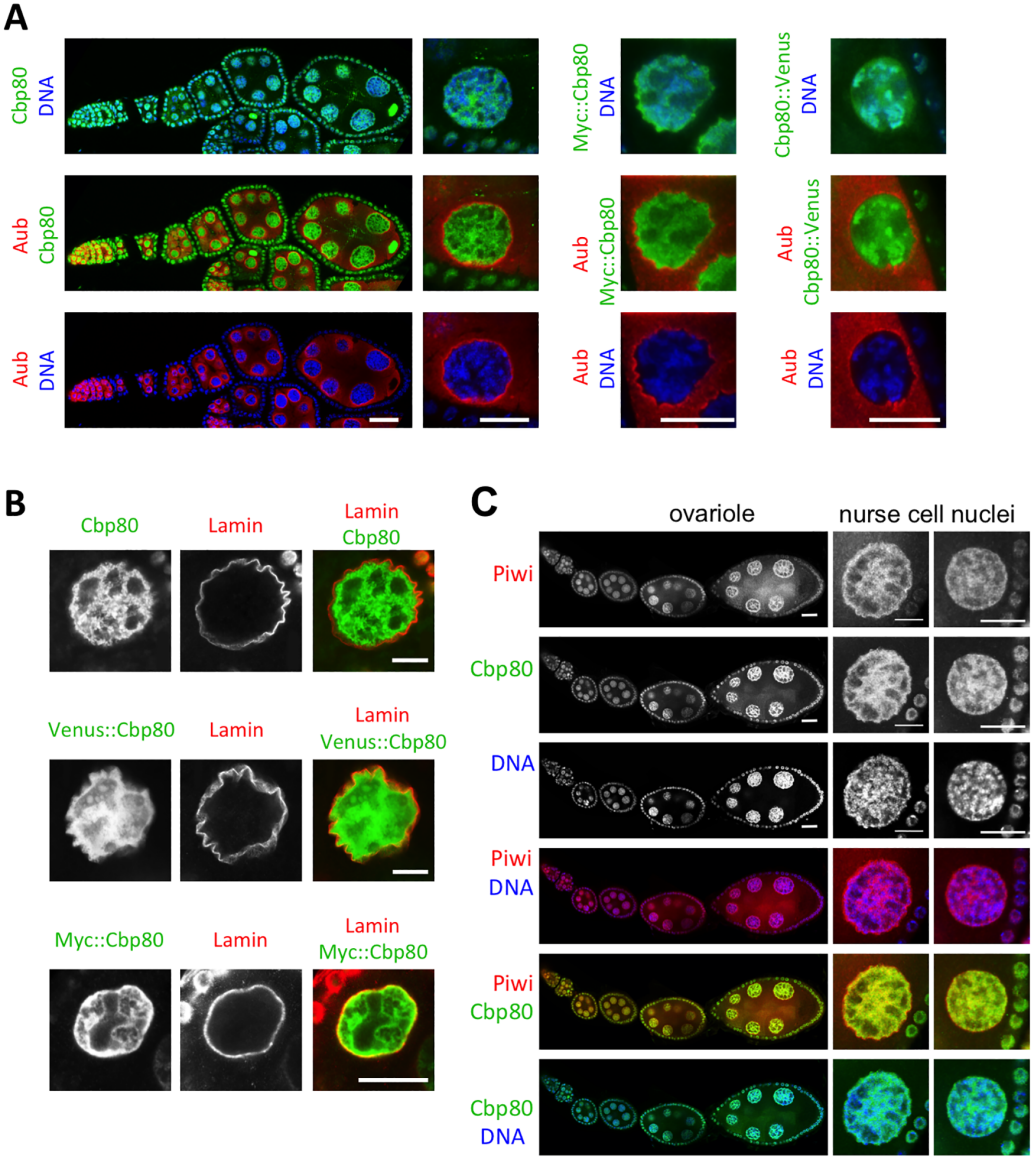
Cbp80 is predominantly nuclear. **(A)** Cbp80 signal (green) was revealed in wild-type ovaries using anti-Cbp80 antibodies. Ovaries expressing a Myc-tagged Cbp80 showed a similar fluorescence signal upon staining with an anti-Myc-epitope antibody. A similar pattern was also observed analyzing the Venus signal directly in ovaries expressing a Venus fusion protein. DNA staining is shown in blue. Anti-Aub staining (red) produces highest signal intensity in the perinuclear nuage with some additional cytoplasmic signal. **(B)** Co-staining for the nuclear envelope protein Lamin and tagged and untagged Cbp80 revealed that the Cbp80 signal is mainly inside the nuclei. **(C)** Cbp80 shows a very similar nuclear distribution pattern as Piwi. Right panels show high magnification of 2 different size nurse cell nuclei.

Most of the processes involving the nCBC take place in the nucleus and in the vicinity of the nuclear envelope. To study the localization of Cbp80 in the germline, we stained wild-type ovaries with anti-Cbp80 antibodies (Fig 3A). Cbp80 protein signal was mainly localized in the nucleus, although some of it was present in the cytoplasm as well. We also generated transgenic flies expressing Myc-tagged Cbp80 using the *GAL4>UASP* system. Overexpressing this construct with the ubiquitously active *actin-GAL4* and *tub-GAL4* drivers did not appear to be toxic because these flies were viable and fertile. The *matα4-GAL4* germline-specific driver was thereafter used to express *UASP-myc*::*Cbp80* in the germline. In addition, we expressed Cbp80 as fusion protein tagged with Venus at the N- and C-term, respectively. We will refer to the tagged fusion proteins as Myc::Cbp80, Venus::Cbp80 and Cbp80::Venus, respectively. Staining *Drosophila* ovaries with tag-specific antibodies and direct assessment of Venus fluorescence showed primarily a nuclear signal in the germline, too (Fig 3A). Cbp80 signal is often seen in the vicinity of the DNA, but also along the nuclear envelope. In order to find out whether the latter localization corresponds to the nuage, a perinuclear organelle that is involved in piRNA production, we also stained these ovaries for the nuage marker Aub (Fig 3A). The three different staining experiments revealed that the Aub signal was peripheral to the Cbp80 signal, suggesting that Cbp80 does not accumulate in "nuages". Staining ovaries of the same genotypes for the nuclear envelope protein Lamin also showed that the intense Cbp80 signal resided within the nucleus, next to the nuclear envelope and the nuclear pores (Fig 3B; S5 Fig). Interestingly, this nuclear Cbp80 distribution pattern in ovaries strongly resembled the one of Piwi (Fig 3C).

### *Cbp80* knockdown affects nuclear localization of Piwi

**Fig 4:**
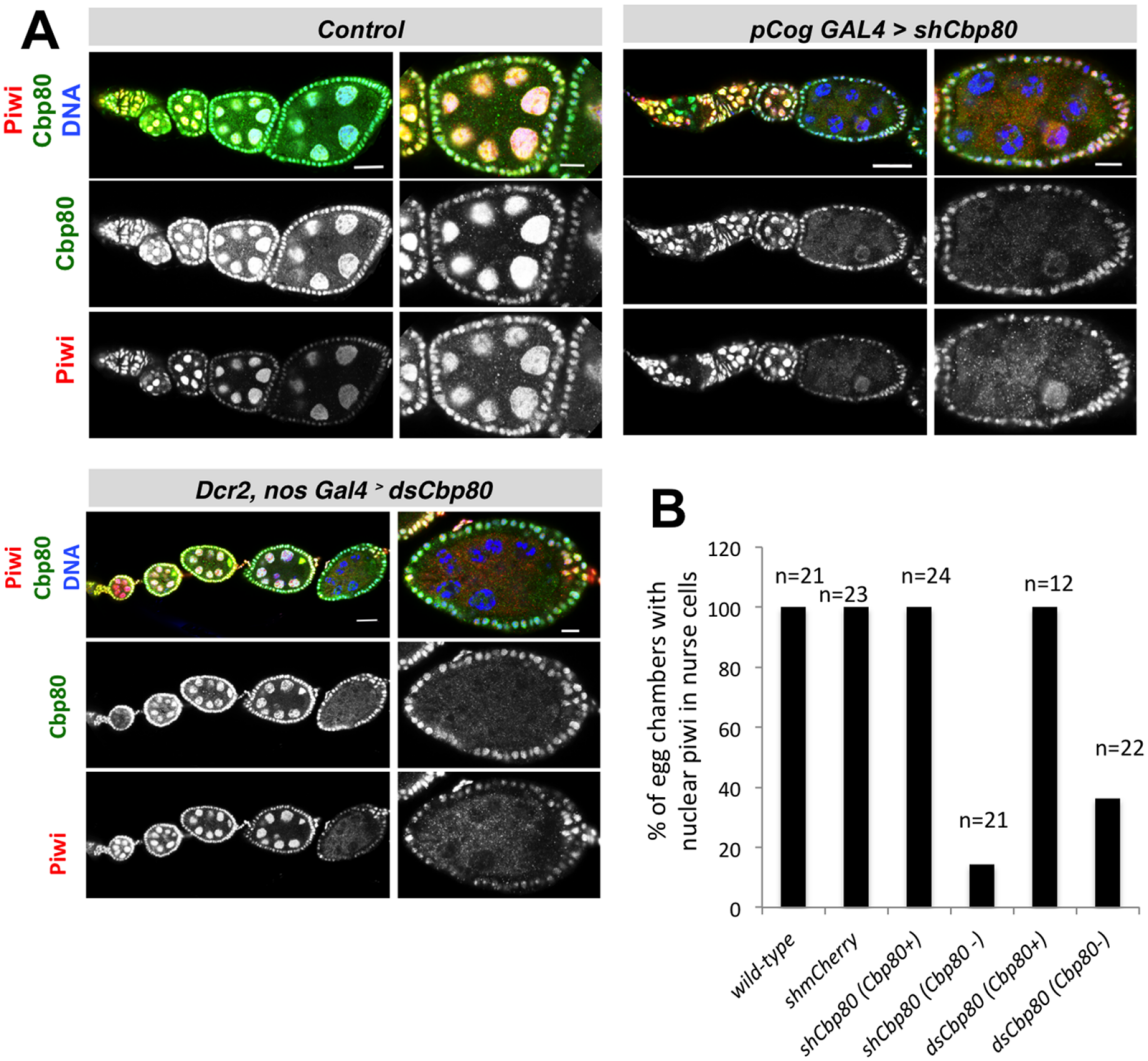
*Cbp80* is needed for nuclear localization of Piwi. **(A)** Germ line knockdown of ovarian *Cbp80*, targetting 2 different parts of *Cbp80*, and control knockdown (*shmCherry*). W*ild-type* egg chambers were an aditional control for quantification. Ovaries showing the "d" phenotype (Fig 1A) upon *Cbp80* knockdown were dissected and co-stained with anti-Cbp80 antibodies (green signal) and anti-Piwi antibodies (red signal). DNA staining (Hoechst) is in blue. Egg chambers with reduced Cbp80 signal in the germline, but normal levels of nuclear Cbp80 signal in the surrounding follicule cells display a healthy normal appearance, but show mis-localization of Piwi protein to the cytoplasm. While rare cases of degenerating or damaged wild-type egg chambers can also show delocalization of Piwi in nurse cells and follicle cells, we did not observe this in healthy wild-type egg chambers. (B) Quantification of nuclear Piwi in the germline where Cbp80 expression is normal (Cbp80+) or efficiently knocked down (Cbp80-, where no or only very weak Cbp80 signal is seen in the germline). Scale bars: 25 μm in all ovariole pictures and 10 μm in magnified egg chamber pictures.

The argonaute sub-family proteins Piwi, Aub and Ago3 play important roles in piRNA-induced silencing. Piwi is normally enriched in the nucleus, a feature reported to be important for its silencing rather than its slicer activity [36] [37]. Aub and Ago3 are cytoplasmic proteins possessing slicer activity and they are key players in the piRNA amplification loop [1] [2] [3] [4]. To learn more about the role of *Cbp80* in the piRNA pathway, we tested the effect of *Cbp80* knockdown on Piwi localization in ovaries. To be able to correlate Piwi expression and distribution with the efficiency of the *Cbp80* knockdown we stained the ovaries simultaneously for Cbp80 and Piwi. To account for staining differences between samples, the nuclear Cbp80 signal in the surrounding somatic follicle cells served as reference signal because we do not expect it to change upon germline specific knock down of *Cbp80*. In wild-type control egg chambers Piwi nuclear localization was clearly detected from early oogenesis stages on (Fig 4A). Upon germline knock down of *Cbp80* we observed that egg chambers that displayed a clearly reduced Cbp80 signal in the germline also showed reduced nuclear localization of Piwi (Fig 4A and 4B). The reduced nuclear levels of Piwi protein were observed upon *Cbp80* knockdown with either *shRNA* or with the long dsRNA targeting different regions of the *Cbp80* mRNA. Furthermore, the strength of the reduction correlated with the reduction of the levels of Cbp80 (Fig 4A and 4B). Interestingly, of the known components of the piRNA pathway, the ones that are needed for the primary piRNA biogenesis pathway are also needed for the nuclear localization of Piwi [7] [6] [28] [38] [39] [40], further supporting the notion that *Cbp80* is essential for piRNA biogenesis.

Given the role of *Cbp80* in the piRNA pathway and in Piwi localization, and the fact that both proteins show similar nuclear accumulation patterns, we also tested for direct interactions between Cbp80 and Piwi. However, yeast 2-hybrid experiments failed to detect a physical interaction between Cbp80 and Piwi (S6 Fig) suggesting that *Cbp80* affects Piwi localization indirectly.

### Normal nuage localization of Aub and Ago3 requires *Cbp80*

**Fig 5:**
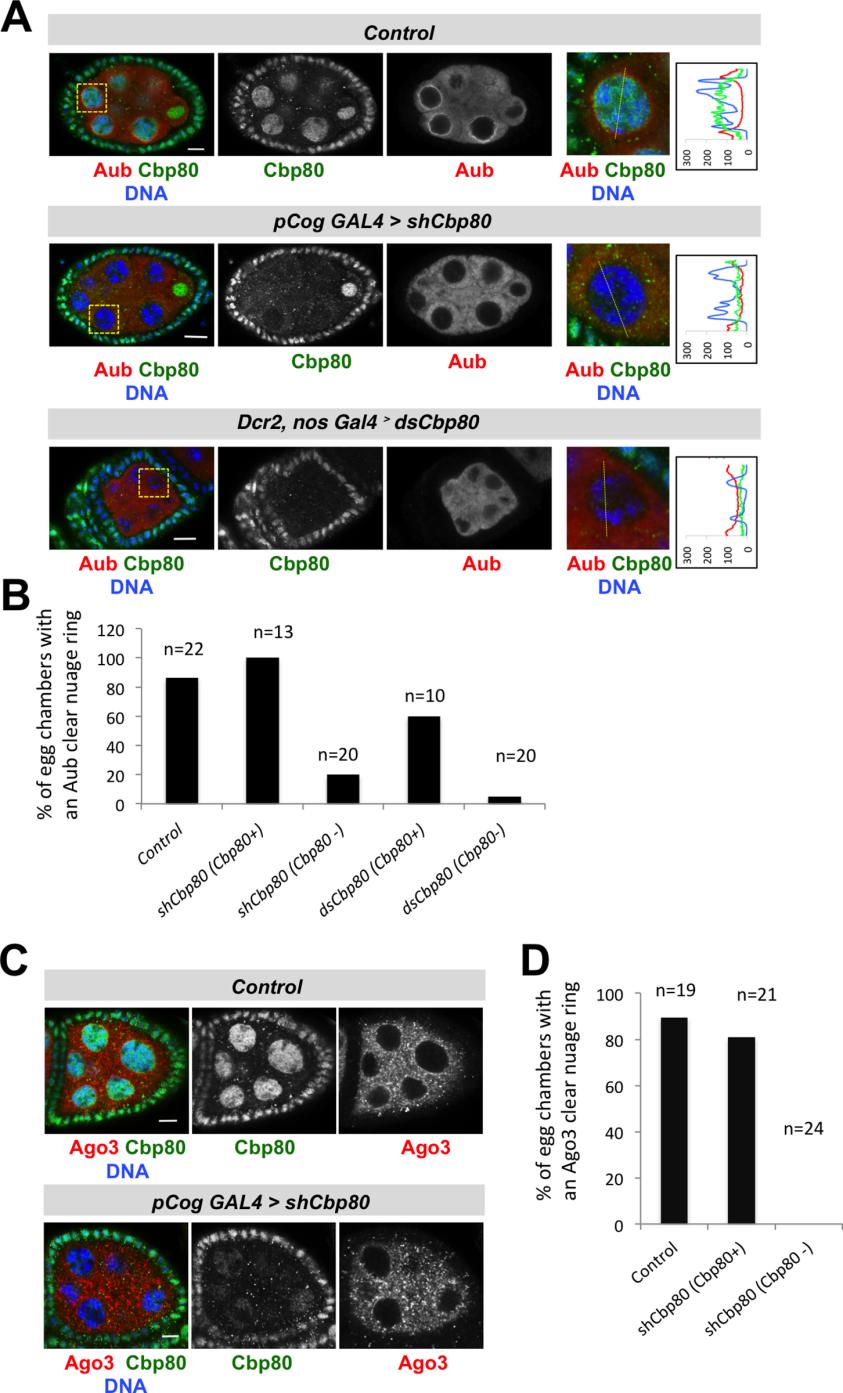
*Cbp80* is needed for enrichment of Aub and Ago3 in the nuage. Germline specific knockdown of *Cbp80* and *mCherry* (control). *Cbp80* knockdown ovaries of the "d" phenotype were used (Fig 1A). Egg chambers were co-stained with anti-Cbp80 antibodies (green signal) and anti-Aub (**A**, red signal), and anti-Ago3 (**C**, red signal), respectively. DNA staining (Hoechst) is in blue. **(A)** The localization of Aub (red), to the nuage is affected by knocking down *Cbp80* with two different RNAi lines (*shCbp80* and *dsCbp80*). The level of Cbp80 knockdown can be judged by comparing the Cbp80 signal in the huge nurse cell nuclei to the signal in the surrounding somatic follicel cell nuclei of the same egg chamber (internal control). This ratio is lower in the knock-down situation than in the controls. Egg chambers with Cbp80 knockdown show no clear nuage ring of Aub staining. Plots on the right of the pictures display the fluorescence signal intensity for each channel across the nurse cell nucleus shown. Scale bars: 7.5 μm in control and 10 μm in *Cbp80* knockdown egg chamber pictures. (B) Quantification of perinuclear Aub accumulation in the germline where Cbp80 expression is normal or only slightly knocked down (Cbp80+), or where it is efficiently knocked down (Cbp80-, where no or only very weak Cbp80 signal is seen in the germline). The presence of the Aub ring in the nuage correlates with the presence of Cbp80. **(C)** In control egg chambers Ago3 (red) signal is slightly concentrated in the immediate vicinity of the nuclear envelope of the nurse cells, the nuage. Upon knock down of *Cbp80*, this localization is reduced or lost. Cbp80 staining (green) reveals efficiency of *Cbp80* knockdown. Scale bars: 7.5μm. **(D)** Quantification of the correlation between Cbp80 levels and Ago3 localization to the nuage as done in B) for Aub localization.

Localization of Aub to the nuage region around nurse cell nuclei was also strongly affected in egg chambers with reduced Cbp80 signal in the germline (Fig 5A and 5B). In control egg chambers the nuage ring of Aub is clearly detected and its signal intensity clearly exceeds the cytoplasmic one (Fig 5A). In contrast, in the germline of most *Cbp80* knockdown egg chambers with lowered Cbp80 signal, we did not observe accumulation of Aub in a ring around the nuclear surface (Fig 5A and 5B). On the other hand, egg chambers expressing the RNAi, but showing no reduction of Cbp80 (due to inefficient knockdown), show mostly a normal localization of Aub (Fig 5B). Because this was observed with both RNAi lines, we conclude that Aub localization to the nuage depends on *Cbp80* function. Similarly, Ago3 enrichment in the immediate vicinity of the nurse cell nuclear envelope was also reduced by the *Cbp80* knockdown (Fig 5C and 5D). The vast majority of control egg chambers and knockdown egg chambers retaining normal levels of nuclear Cbp80 showed a clear signal of Ago3 as a ring around the nurse cell nuclei. In contrast, egg chambers with reduced levels of germline Cbp80 show no discernable perinuclear ring of Ago3 signal even though expression of Ago3 was detectable in the cytoplasm of these egg chambers (Fig 5C and 5D). The fact that reducing levels of Cbp80 also affects the localization of Ago3 and Aub may also explain the requirement for *Cbp80* for the ping-pong amplification pathway, which is less dependent on Piwi, but depends on *Ago3* and *aub* [41] [3].

### Effect of reduced *Cbp80* on levels of RNAs and proteins involved in piRNA biogenesis

Because *Cbp80* plays a role in transcription, stability and nuclear export of RNAs, we considered the possibility that *Cbp80* is primarily involved in piRNA precursor biogenesis and that defects at this step could lead to mislocalization of Piwi protein. We performed RNA *in situ* experiments using Stellaris probes designed to detect the piRNA precursors for the *42AB* and the *20A* clusters (S7 Fig; [13]). In order to be able to correlate possible phenotypes with the reduction of Cbp80 levels, we adopted the protocol for simultaneous labeling of RNAs by in situ hybridization and labeling of proteins by antibody staining. Precursor transcripts were mainly detected inside the nuclei and in the perinuclear nuage region where their processing takes place. To be able to discriminate between these two compartments we also co-stained the ovaries with Lamin. Clear blobs of cluster staining in the nurse cell nuclei were observed even in the absence of Cbp80, arguing that the transcription and stability of precursors transcripts is not strongly affected upon *Cbp80* knockdown (S7 Fig). Confirming these results by quantitative RT-PCR, we found that precursor transcript levels from two regions of cluster *42AB* (region A and 1–32) were also not reduced upon *Cbp80* knockdown (S8 Fig). By analyzing the subcellular localization of the transcript precursors, we observed very few dots of the *42AB* and *20A* probes outside the nuclei in the nuage region and larger blobs in the nuclei where the transcripts are clustered at the site of transcription (S7 Fig). The signal, at least for the *42AB* probe was specific since it disappeared in egg chambers where *Rhino* (*Rhi*) was knocked down and *Rhi* is essential for transcription of this dual strand cluster (S7 Fig; [13]). The low levels of precursor RNAs in the nuage are probably due to the fact that their nuclear export and processing are tightly coupled and every precursor transcript gets immediately chopped up when exiting the nucleus. Cytoplasmic (nuage) precursors would then not be abundant enough to quantify an effect of *Cbp80* knockdown on precursor RNA export. In any case, trying to quantify these effects we did not observe a clear reduction of the nuage signals. In summary, although we cannot rule out an effect on piRNA precursor export, the nuclear export of the piRNA precursors is clearly not completely abolished upon *Cbp80* knockdown, (S7 Fig).

**Fig 6:**
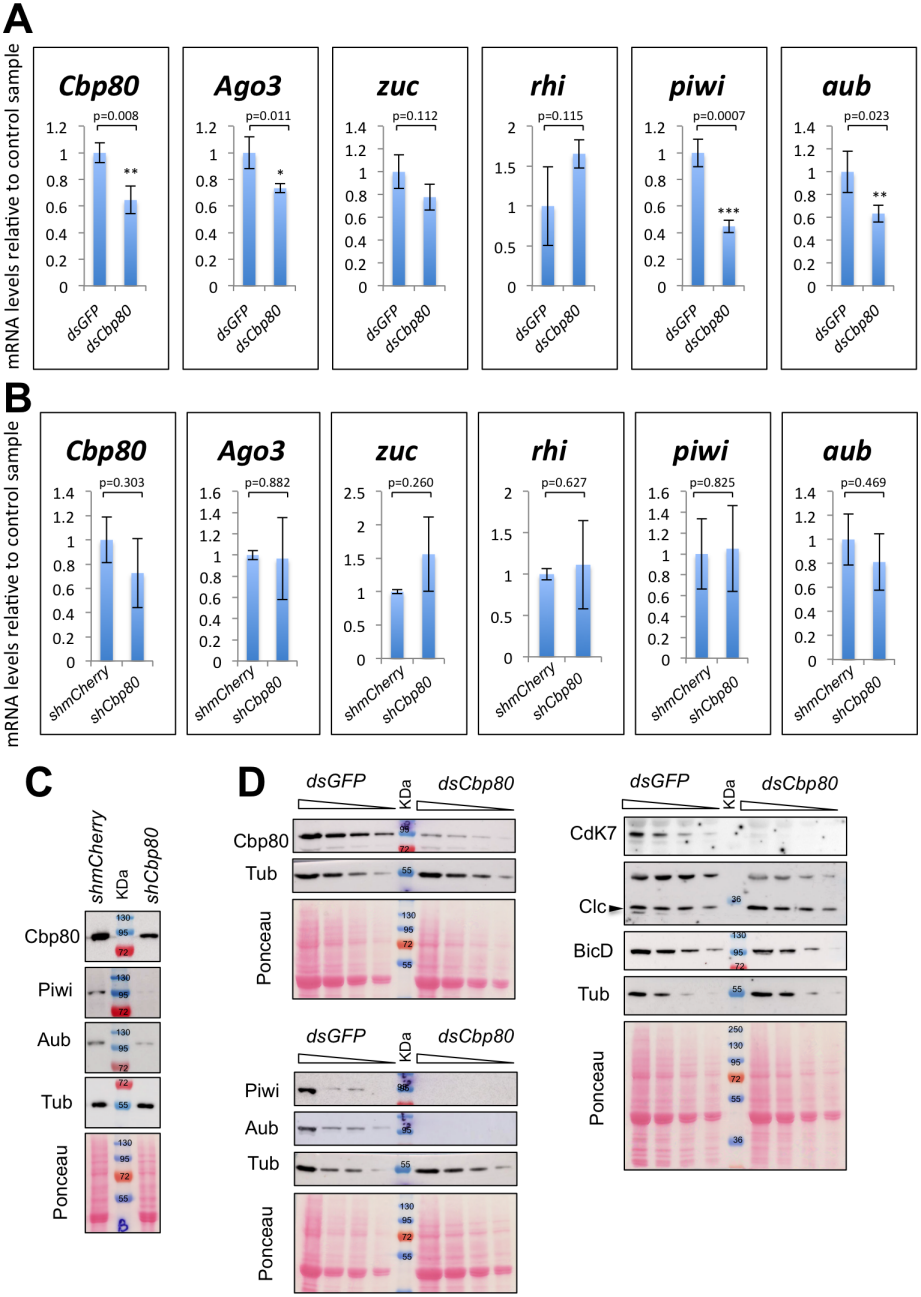
Expression of piRNA pathway components requires *Cbp80*. **(A, D)** Knock down of *Cbp80* in egg chambers expressing a *dsRNA* against *Cbp80 (dsCbp80*) driven by the GAL4-nos.NGT40 driver and the UAS-Dcr2. Control flies expressed a *dsRNA* against *GFP* (*dsGFP*) **(B, C)** Ovaries expressing specifically in the germline (*pCog-Gal4* driver) shRNAs against *Cbp80* or *mCherry* (as control). Control flies used for RT-PCR experiments expressed also a Jupiter-mCherry fusion protein. **(A-B)** mRNA levels of piRNA pathway factors (*piwi*, *aub*, *Rhi*, *zuc* and *Ago3*) and *Cbp80* were measured by qRT-PCR. Fold expression levels of each mRNA in the knockdown samples relative to its expression in the corresponding control are shown for each mRNA. Total starting RNA amounts were the same in both samples. Error bars represent ^+^/- SD of 2 controls in B and 3 control samples in A and 3 biological knockdown replicates. *p<0.05; **p<0.01; ***p<0.001. **(C-D)** Levels of Cbp80, Piwi, Aub, BicD, Cdk7, Clc and Tub (as loading control) were assessed by Western blotting. Ponceau staining is also shown to reveal total proteins as loading control. Levels Piwi, Aub and Cdk7 were strongly reduced upon *Cbp80* reduction. On the other hand, levels of Tub, Clc and BicD were less affected. For Cbp80 knockdown samples were extracted from egg chambers showing the phenotype “d” (Fig 1A).

We also considered the possibility, that *Cbp80* may be needed for the normal expression of mRNAs encoding protein components of the Piwi pathway. Because *Cbp80* might be needed for the stability and transcription of many mRNAs, including the ones coding for subunits of RNA Pol1, 2 and 3, we standardized specific mRNA levels measured by qPCR to total RNA levels. In this way mRNA levels in the *Cbp80* knockdown were compared to the control samples (Fig 6A). In addition, we normalized the levels of Piwi component mRNAs with the set of mRNAs used previously (S9 Fig). Germline knock down with the dsRNA against *Cbp80* consistently led to a significant reduction of piwi mRNA regardless of whether normalization was performed with total RNA levels or the different control mRNAs (Fig 6A and S9A Fig). A significant reduction of the mRNA levels for other Piwi components (Ago3 and Aub) was also observed when results were normalized to total RNA levels, but their reduction was less clear when specific genes were used for normalization (Fig 6A and S9A Fig). In contrast to these results, knocking down *Cbp80* with the shRNA had no clear effects on the levels of Piwi component mRNAs (Fig 6B and S9B Fig). The variability might be due to differential effects of *Cbp80* knockdown on the expression of individual genes.

Even if the effect on mRNA levels of Piwi components is mild, we clearly observed that levels of Piwi and Aub proteins were strongly reduced upon knockdown of *Cbp80* (Fig 6C and 6D). Furthermore, as seen in two independent knockdown assays, these proteins were clearly reduced when normalized to Tubulin or to total protein levels (Fig 6C and 6D). Interestingly, levels of Cyclin dependent kinase 7 (Cdk7) were also strongly reduced upon *Cbp80* knockdown and Piwi proteins had previously been implicated in the upregulation of the expression of Cdks and Cyclins and in enhancing cell proliferation in several cancers [42–45]. In contrast to Piwi and Aub proteins, levels of Bicaudal-D (BicD) and Clathrin light chain (Clc), two proteins unrelated to the Piwi pathway, were only mildly affected by the *Cbp80* knockdown (Fig 6D). The effect on them was similar to the effect on the Tubulin control. The stronger effect on the levels of Piwi proteins suggests that *Cbp80* affects piRNA production most likely through its requirement for the correct expression and normal localization of these protein factors of the Piwi pathway.

## Discussion

Knocking down *Cbp80* with a set of different germline-specific drivers produced a variety of phenotypes. *Cbp80* is clearly essential for germline survival because its knockdown can cause the formation of rudimentary ovaries that lack germ cells. Surprisingly, however, the requirement for germline development and survival seemed to be restricted to the early, the germarial stages of oogenesis. *Gal4* drivers that drive *Cbp80* RNAi expression during the germarial stages, and uniquely these drivers, blocked germline development efficiently. For instance, the *nos-Gal4* driver is expressed during early stages of germarial development and it caused an early and almost complete disappearance of the germline. The *pCog-Gal4* driver promoted high expression levels of genes under UASP control in the later phase of germarial development (late in region 2 and in region 3), and the knockdown of *Cbp80* with this driver caused the formation of rudimentary ovaries, but still allowed many egg chambers to develop to stage 8 or 9, and some even further. In contrast, the *matα4-GAL4* driver is a very strong germline driver, but it is only active slightly later in ovarian development, in the vitellarial stages. Surprisingly, even this strong driver did not cause any morphologically visible mutant phenotypes when it was used to knock down *Cbp80*. Germline cells therefore seem to have a particularly high need for Cbp80 during the period when *nos-Gal4* and *pCog-Gal4* are expressed in the germarium, but they seem to be less dependent on Cbp80 for their development and survival during the later phases when *matα4-GAL4* is active. Interestingly, a stage-specific requirement for piRNA pathway genes has also been reported by others [46]. These authors found that *aub*, *vasa* and *spn-E* are necessary in very early stages of oogenesis within the germarium, but they appear to become less important for efficient TE silencing in later stages (whereas *piwi*, *ago3*, *mael* appear to be required throughout oogenesis). The requirement for these genes seems to coincide with a period of restarting the piRNA production after the germ cells have moved beyond the stage with reduced Piwi, called the ‘Piwiless pocket’ [46]. Coinciding with this overlapping stage specific requirement for *Cbp80* and the genes involved in the production of the protein components of the piRNA pathway, we also observed that the expression levels (and localization) of Piwi and Aub protein depends much more strongly on *Cbp80* than the expression of control proteins unrelated to the Piwi pathway. While we do not know the basis of this differential requirement, and this represents one of the limitations of this study, this differential effect seems to argue against a random pleiotropic effect.

Several groups provided evidence that in *Drosophila* noncanonical transcription coupled with splicing- and termination inhibition discriminates piRNA precursors from mRNAs and ensures their correct processing [13] [47] [48]. The current model proposes that dual-strand cluster transcription is achieved by read-through transcription from convergent neighboring genes or by noncanonical transcription initiation by RNA polymerase II (Pol II). In both situations, piRNA-mediated recruitment of Piwi to a dual-strand cluster locus leads to H3K9 trimethylation and the subsequent recruitment of the RDC complex consisting of Rhi, Deadlock (Del) and Cuttoff (Cuff). The cited authors also proposed models in which the upstream transcript undergoes 3′ end processing and the binding of the RDC complex to chromatin brings it in close proximity to the newly formed 5′ end of a nascent piRNA precursor transcript. The binding of the RDC complex then prevents processing and degradation of the transcript. This step would distinguish piRNA precursors from mRNAs, which are bound by CBC, spliced, exported and subsequently bound by eIF4E that promotes translation initiation. It is interesting to analyze our results in the context of this model. *Cbp80* knockdown did not significantly alter the expression of piRNA precursors from the dual strand cluster *42AB* and it even led to higher expression of transposable elements. In contrast, *Cbp80* knockdown interfered with expression and localization of the protein components of the piRNA pathway. It therefore seems that in the piRNA biogenesis pathway *Cbp80* functions mainly in the production and localization of the protein components of the piRNA pathway because these factors differentially dependent on *Cbp80*.

## Supporting information

S1 Fig Expression pattern of germline-specific GAL4 drivers. pUASP-Venus::Cbp80 tagged fly lines were crossed to different germline-specific GAL4 drivers. Ovaries from the resulting flies were dissected and DNA was stained with Hoechst. Venus signal is mostly nuclear. Scale bar: 25 μm.
(PDF)

S2 Fig Cbp80 protein levels correlate with the severity of the different phenotypes observed upon Cbp80 knockdown. Ovaries expressing specifically in the germline (pCog-Gal4 driver) shRNAs against *Cbp80* or *mCherry* (as control) were used. Ovaries showing normal appearance upon *Cbp80* knockdown (“n” phenotype; Fig 1), underdeveloped ovaries ("d" phenotype; Fig 1) and control ovaries were tested for Cbp80 levels by Western blotting. Tubulin was used as a loading control. 2 different amounts of each *Cbp80* knockdown sample were loaded.
(PDF)

S3 Fig Upregulation of transposons (TEs) upon *Cbp80* knockdown. Ovaries displaying the "d" phenotype upon *Cbp80* knockdown were used in all experiments. (A-C) Fold increase in RNA levels of indicated TEs upon germline-specific RNAi-mediated knock down of *Cbp80* (shRNA against *Cbp80*). The germline GAL4 driver alone was used as control. (A-B) Fold-changes in transposon RNA levels were normalized to *rp49*, *Tub* and *BicD* levels. Control ovaries expressed the *shmCherry* construct. (C) Levels of transposon transcripts relative to the control sample are shown. The same amount of total RNA was used as starting material. Error bars represent ^+^/- SD of 2 control and 3 biological knock down replicates. (D-F) Fold increase in RNA levels of the same TEs upon germ line specific knock down of *Cbp80* using *dsRNA*. (D-E) Fold changes relative to *rp49*, *Tub* and *BicD*. Control ovaries expressed a *dsGFP* RNAi construct. (F) Levels of transposon transcripts relative to the control sample are shown. The same amount of total RNA was used as starting material. Error bars represent ^+^/- SD of 3 biological replicates. *p<0.05; **p<0.01; ***p<0.001.
(PDF)

S4 Fig Germline *Cbp80* is involved in producing piRNAs derived from the *20A* cluster. Genotypes of ovaries analyzed are depicted on top of the figures. Histogram showing small RNAs (23–29 nucleotides long) mapping to the germline-specific uni-strand cluster *20A* in flies expressing specifically in their germline shRNAs against *Cbp80* or *mCherry* (as control). The germ line specific *pCog-Gal4* driver was used for their expression.
(PDF)

S5 Fig Tagged and untagged Cbp80 show similar subcellular accumulation patterns. Cbp80 signal (green) is primarily seen inside the nucleus in *Drosophila* nurse cells. Nuclear compartments are delineated by the nuclear envelope protein Lamin (red). Single nurse cell nuclei are shown and the DNA is stained in blue. Scale bar: 10 μm.
(PDF)

S6 Fig Piwi does not interact directly with Cbp80 in the yeast two-hybrid system. Interaction test of Cbp80 either in the DNA binding domain (*BD*, *upper*) or in the activator domain (*AD*, lower) vector with Piwi. Cbp20 and empty vectors were used as positive and negative controls, respectively. No interaction between Cbp80 and Piwi was detected in either case.
(PDF)

S7 Fig Export of piRNA precursors is not significantly affected by *Cbp80* knockdown. (A-B) Ovaries expressing specifically in the germline (pCog-Gal4 driver) shRNAs against *Cbp80* or *mCherry* (as control). For the *Cbp80* knockdown, only partially developed ovaries were collected. Ovarioles were stained at the same time for Lamin (blue), Cbp80 (green) and the piRNA precursor transcripts from clusters *42AB* (red) and *20A* (green). Left pictures show confocal images of nurse cell nuclei stained for Lamin and Cbp80. Right pictures show the signals for Lamin and the transcripts. Upper panels show an example of a control egg chamber with a clear nuclear Cbp80 signal. Lower panels show a *Cbp80* knockdown example with strongly reduced nuclear Cbp80 staining. Levels and localization of the *42AB* and *20A* cluster transcripts show no clear change upon *Cbp80* knockdown. (B) Anti-Lamin staining allowed us to classify perinuclear dots from the *42AB* and *20A* transcripts as residing in the ‘nuage’ region (if they were within approx. 1μm of the Lamin signal) or inside the nucleus. Dots overlapping with the Lamin signal were not counted. The percentage of transcripts in the ‘nuage’ (relative to transcripts in the ‘nuage’ and the nucleus) was ploted for control and *Cbp80* knockdown. No significant differences were observed between them. (C) Ovaries expressing specifically in the germline (MTD-Gal4 driver) shRNAs against *Rhi* or *white* (as control) were used to test the specificity of the *42AB* probe. Ovarioles were stained at the same time for Lamin, Rhi and the *42AB* and *20A* piRNA precursor transcripts. Left pictures show confocal images of nurse cell nuclei stained for Lamin (blue) and Rhi (green). Right pictures show the signals for Lamin (blue), *42AB* transcripts (red) and *20A* transcripts (green). The signal for the *42AB* probe was lost upon Rhi knockdown, which affects transcription from this cluster, confirming the specificity of the probe and the *in situ* protocol used. Expression of the *20A* cluster is not affected (as expected).
(PDF)

S8 Fig Levels of *42AB* precursor transcripts are not reduced upon *Cbp80* knockdown. Ovaries expressed in their germ line shRNAs against *Cbp80 (trip line*) and shRNAs against *mCherry* (as control), respectively, under the pCog-Gal4 driver. Control flies expressed also a *Jupiter-mCherry* fusion gene in a *jupiter*^+^ background. Levels of precursor transcripts for two regions of the *42AB* cluster, regions *A* and *1–32*, and the *flam* locus were measured by qRT-PCR. Fold expression levels relative to the expression of *rp49* are shown for each sample. Error bars represent ^+^/-SD of 2 control and 3 biological knock down replicates. While there is a high variability between the different biological samples (probably due to the phenotypic differences between the knock down samples and the size-matched wild-type stages), no reduction in the expression of piRNA precursors was observed upon *Cbp80* knockdown.
(PDF)

S9 Fig mRNAs coding for piRNA pathway components display different sensitivities to *Cbp80* knockdown. (A) Ovaries expressing specifically in the germline (pCog-Gal4 driver) shRNAs against *Cbp80 (shCbp80)* or *mCherry* (*shmCherry*; as control) were used. Control flies expressed also a Jupiter-mCherry fusion protein. (B) Egg chambers expressing a *dsRNA* against *Cbp80 (dsCbp80*) under the control of the GAL4-nos.NGT40 driver combined with the *UAS-Dcr2*. Control flies expressed a *dsRNA* against *GFP* (*dsGFP*). (A-B) Ovaries showing the "d" phenotype (Fig 1) upon *Cbp80* knockdown were used. mRNA levels of piRNA pathway factors were tested by qRT-PCR. The expression of the piwi component mRNAs was normalized relative to the expression of control genes (*Tub*, *BicD* and *rp49*). Error bars represent ^+^/- SD of 2 control (in B) and 3 control samples (in A) and 3 biological knock down replicates. *p<0.05; **p<0.01; ***p<0.001.
(PDF)

## References

[1] Siomi MC, Sato K, Pezic D, Aravin AA. PIWI-interacting small RNAs: the vanguard of genome defence. Nature reviews Molecular cell biology. Nature Publishing Group; 2011;12: 246–258. doi: 10.1038/nrm3089 21427766 10.1038/nrm3089

[2] Czech B, Hannon GJ. One Loop to Rule Them All: The Ping-Pong Cycle and piRNA-Guided Silencing. Trends Biochem Sci. 2016;41: 324–337. doi: 10.1016/j.tibs.2015.12.008 26810602 10.1016/j.tibs.2015.12.008 PMC4819955

[3] Brennecke J, Aravin AA, Stark A, Dus M, Kellis M, Sachidanandam R, et al Discrete small RNA-generating loci as master regulators of transposon activity in Drosophila. Cell. 2007 ed. 2007;128: 1089–1103. doi: 10.1016/j.cell.2007.01.043 17346786 10.1016/j.cell.2007.01.043

[4] Gunawardane LS, Saito K, Nishida KM, Miyoshi K, Kawamura Y, Nagami T, et al A slicer-mediated mechanism for repeat-associated siRNA 5' end formation in Drosophila. Science. 2007;315: 1587–1590. doi: 10.1126/science.1140494 17322028 10.1126/science.1140494

[5] Schupbach T, Wieschaus E. Female sterile mutations on the second chromosome of Drosophila melanogaster.—I. Maternal effect mutations. Genetics. 1989;121: 101–117. 2492966 10.1093/genetics/121.1.101 PMC1203592

[6] Preall JB, Czech B, Guzzardo PM, Muerdter F, Hannon GJ. shutdown is a component of the Drosophila piRNA biogenesis machinery. RNA. Cold Spring Harbor Lab; 2012;18: 1446–1457. doi: 10.1261/rna.034405.112 22753781 10.1261/rna.034405.112 PMC3404366

[7] Czech B, Preall JB, McGinn J, Hannon GJ. A transcriptome-wide RNAi screen in the Drosophila ovary reveals factors of the germline piRNA pathway. Molecular cell. 2013;50: 749–761. doi: 10.1016/j.molcel.2013.04.007 23665227 10.1016/j.molcel.2013.04.007 PMC3724427

[8] Handler D, Meixner K, Pizka M, Lauss K, Schmied C, Gruber FS, et al The genetic makeup of the Drosophila piRNA pathway. Molecular cell. 2013;50: 762–777. doi: 10.1016/j.molcel.2013.04.031 23665231 10.1016/j.molcel.2013.04.031 PMC3679447

[9] Muerdter F, Guzzardo PM, Gillis J, Luo Y, Yu Y, Chen C, et al A genome-wide RNAi screen draws a genetic framework for transposon control and primary piRNA biogenesis in Drosophila. Molecular cell. 2013;50: 736–748. doi: 10.1016/j.molcel.2013.04.006 23665228 10.1016/j.molcel.2013.04.006 PMC3724422

[10] Topisirovic I, Svitkin YV, Sonenberg N, Shatkin AJ. Cap and cap-binding proteins in the control of gene expression. Wiley interdisciplinary reviews RNA. 2011 ed. 2011;2: 277–298. doi: 10.1002/wrna.52 21957010 10.1002/wrna.52

[11] Bischof J, Maeda RK, Hediger M, Karch F, Basler K. An optimized transgenesis system for Drosophila using germ-line-specific phiC31 integrases. Proceedings of the National Academy of Sciences of the United States of America. 2007;104: 3312–3317. doi: 10.1073/pnas.0611511104 17360644 10.1073/pnas.0611511104 PMC1805588

[12] Koch R, Ledermann R, Urwyler O, Heller M, Suter B. Systematic functional analysis of Bicaudal-D serine phosphorylation and intragenic suppression of a female sterile allele of BicD. PloS one. 2009 ed. 2009;4: e4552 doi: 10.1371/journal.pone.0004552 19234596 10.1371/journal.pone.0004552 PMC2639643

[13] Mohn F, Sienski G, Handler D, Brennecke J. The rhino-deadlock-cutoff complex licenses noncanonical transcription of dual-strand piRNA clusters in Drosophila. Cell. 2014;157: 1364–1379. doi: 10.1016/j.cell.2014.04.031 24906153 10.1016/j.cell.2014.04.031

[14] Rorth P. Gal4 in the Drosophila female germline. Mechanisms of Development. 1998 ed. 1998;78: 113–118. 9858703 10.1016/s0925-4773(98)00157-9

[15] Vazquez-Pianzola P, Urlaub H, Suter B. Pabp binds to the osk 3'UTR and specifically contributes to osk mRNA stability and oocyte accumulation. Developmental biology. 2011 ed. 2011;357: 404–418. doi: 10.1016/j.ydbio.2011.07.009 21782810 10.1016/j.ydbio.2011.07.009

[16] Nag R. Mms19 and xpd: modulators of mitotic kinases in Drosophila. Suter B, editor. 2016.

[17] Vazquez-Pianzola P, Adam J, Haldemann D, Hain D, Urlaub H, Suter B. Clathrin heavy chain plays multiple roles in polarizing the Drosophila oocyte downstream of Bic-D. Development. The Company of Biologists Limited; 2014;141: 1915–1926. doi: 10.1242/dev.099432 24718986 10.1242/dev.099432

[18] Saito K, Nishida KM, Mori T, Kawamura Y, Miyoshi K, Nagami T, et al Specific association of Piwi with rasiRNAs derived from retrotransposon and heterochromatic regions in the Drosophila genome. Genes & development. Cold Spring Harbor Lab; 2006;20: 2214–2222. doi: 10.1101/gad.1454806 16882972 10.1101/gad.1454806 PMC1553205

[19] Nishida KM, Saito K, Mori T, Kawamura Y, Nagami-Okada T, Inagaki S, et al Gene silencing mechanisms mediated by Aubergine piRNA complexes in Drosophila male gonad. RNA. Cold Spring Harbor Lab; 2007;13: 1911–1922. doi: 10.1261/rna.744307 17872506 10.1261/rna.744307 PMC2040086

[20] Sato K, Iwasaki YW, Shibuya A, Carninci P, Tsuchizawa Y, Ishizu H, et al Krimper Enforces an Antisense Bias on piRNA Pools by Binding AGO3 in the Drosophila Germline. Molecular cell. 2015;59: 553–563. doi: 10.1016/j.molcel.2015.06.024 26212455 10.1016/j.molcel.2015.06.024

[21] Gurskiy D, Orlova A, Vorobyeva N, Nabirochkina E, Krasnov A, Shidlovskii Y, et al The DUBm subunit Sgf11 is required for mRNA export and interacts with Cbp80 in Drosophila. Nucleic acids research. Oxford University Press; 2012;40: 10689–10700. doi: 10.1093/nar/gks857 22989713 10.1093/nar/gks857 PMC3510517

[22] Stuurman N, Maus N, Fisher PA. Interphase phosphorylation of the Drosophila nuclear lamin: site-mapping using a monoclonal antibody. Journal of cell science. 1995;108 (Pt 9): 3137–3144. 8537453 10.1242/jcs.108.9.3137

[23] Cagney G, Uetz P, Fields S. High-throughput screening for protein-protein interactions using two-hybrid assay. Methods in enzymology. 2000;328: 3–14. 11075334 10.1016/s0076-6879(00)28386-9

[24] Klattenhoff C, Xi H, Li C, Lee S, Xu J, Khurana JS, et al The Drosophila HP1 homolog Rhino is required for transposon silencing and piRNA production by dual-strand clusters. Cell. 2009;138: 1137–1149. doi: 10.1016/j.cell.2009.07.014 19732946 10.1016/j.cell.2009.07.014 PMC2770713

[25] Livak KJ, Schmittgen TD. Analysis of relative gene expression data using real-time quantitative PCR and the 2(-Delta Delta C(T)) Method. Methods. 2001;25: 402–408. doi: 10.1006/meth.2001.1262 11846609 10.1006/meth.2001.1262

[26] Box G. E. P., Cox DR. An analysis of transformations. Journal of the Royal Statistical Society Series B-Methodological. 1964;26: 211–252.

[27] Spitzer JT. A primer on box-cox estimation. The Review of Economics and Statistics. 1982;64: 307–313.

[28] Olivieri D, Sykora MM, Sachidanandam R, Mechtler K, Brennecke J. An in vivo RNAi assay identifies major genetic and cellular requirements for primary piRNA biogenesis in Drosophila. EMBO Journal. 2010;29: 3301–3317. doi: 10.1038/emboj.2010.212 20818334 10.1038/emboj.2010.212 PMC2957214

[29] Suter B, Steward R. Requirement for phosphorylation and localization of the Bicaudal-D protein in Drosophila oocyte differentiation. Cell. 1991;67: 917–926. 1959135 10.1016/0092-8674(91)90365-6

[30] Larochelle S, Pandur J, Fisher RP, Salz HK, Suter B. Cdk7 is essential for mitosis and for in vivo Cdk-activating kinase activity. Genes & development. 1998 ed. 1998;12: 370–381. 9450931 10.1101/gad.12.3.370 PMC316490

[31] Heerssen H, Fetter RD, Davis GW. Clathrin dependence of synaptic-vesicle formation at the Drosophila neuromuscular junction. Current Biology. 2008;18: 401–409. doi: 10.1016/j.cub.2008.02.055 18356056 10.1016/j.cub.2008.02.055 PMC2699046

[32] Bolger AM, Lohse M, Usadel B. Trimmomatic: a flexible trimmer for Illumina sequence data. Bioinformatics. Oxford University Press; 2014;30: 2114–2120. doi: 10.1093/bioinformatics/btu170 24695404 10.1093/bioinformatics/btu170 PMC4103590

[33] Langmead B, Trapnell C, Pop M, Salzberg SL. Ultrafast and memory-efficient alignment of short DNA sequences to the human genome. Genome Biol. BioMed Central Ltd; 2009;10: R25 doi: 10.1186/gb-2009-10-3-r25 19261174 10.1186/gb-2009-10-3-r25 PMC2690996

[34] Staller MV, Yan D, Randklev S, Bragdon MD, Wunderlich ZB, Tao R, et al Depleting gene activities in early Drosophila embryos with the “maternal-Gal4-shRNA” system. Genetics. Genetics; 2013;193: 51–61. doi: 10.1534/genetics.112.144915 23105012 10.1534/genetics.112.144915 PMC3527254

[35] Ni JQ, Zhou R, Czech B, Liu L. P., Holderbaum L, Yang-Zhou D, et al A genome-scale shRNA resource for transgenic RNAi in Drosophila. Nat Methods. 2011 ed. 2011;8: 405–407. doi: 10.1038/nmeth.1592 21460824 10.1038/nmeth.1592 PMC3489273

[36] Saito K, Ishizu H, Komai M, Kotani H, Kawamura Y, Nishida KM, et al Roles for the Yb body components Armitage and Yb in primary piRNA biogenesis in Drosophila. Genes & development. 2010 ed. Cold Spring Harbor Lab; 2010;24: 2493–2498. doi: 10.1101/gad.1989510 20966047 10.1101/gad.1989510 PMC2975925

[37] Klenov MS, Sokolova OA, Yakushev EY, Stolyarenko AD, Mikhaleva EA, Lavrov SA, et al Separation of stem cell maintenance and transposon silencing functions of Piwi protein. Proceedings of the National Academy of Sciences of the United States of America. 2011 ed. 2011;108: 18760–18765. doi: 10.1073/pnas.1106676108 22065765 10.1073/pnas.1106676108 PMC3219103

[38] Haase AD, Fenoglio S, Muerdter F, Guzzardo PM, Czech B, Pappin DJ, et al Probing the initiation and effector phases of the somatic piRNA pathway in Drosophila. Genes & development. Cold Spring Harbor Lab; 2010;24: 2499–2504. doi: 10.1101/gad.1968110 20966049 10.1101/gad.1968110 PMC2975926

[39] Olivieri D, Senti K-A, Subramanian S, Sachidanandam R, Brennecke J. The cochaperone shutdown defines a group of biogenesis factors essential for all piRNA populations in Drosophila. Molecular cell. 2012;47: 954–969. doi: 10.1016/j.molcel.2012.07.021 22902557 10.1016/j.molcel.2012.07.021 PMC3463805

[40] Li C, Vagin VV, Lee S, Xu J, Ma S, Xi H, et al Collapse of germline piRNAs in the absence of Argonaute3 reveals somatic piRNAs in flies. Cell. 2009 ed. 2009;137: 509–521. doi: 10.1016/j.cell.2009.04.027 19395009 10.1016/j.cell.2009.04.027 PMC2768572

[41] Malone CD, Brennecke J, Dus M, Stark A, McCombie WR, Sachidanandam R, et al Specialized piRNA pathways act in germline and somatic tissues of the Drosophila ovary. Cell. 2009;137: 522–535. doi: 10.1016/j.cell.2009.03.040 19395010 10.1016/j.cell.2009.03.040 PMC2882632

[42] Qu X, Liu J, Zhong X, Li X, Zhang Q. PIWIL2 promotes progression of non-small cell lung cancer by inducing CDK2 and Cyclin A expression. J Transl Med. BioMed Central; 2015;13: 301 doi: 10.1186/s12967-015-0666-y 26373553 10.1186/s12967-015-0666-y PMC4571108

[43] Lee JH, Jung C, Javadian-Elyaderani P, Schweyer S, Schütte D, Shoukier M, et al Pathways of proliferation and antiapoptosis driven in breast cancer stem cells by stem cell protein piwil2. Cancer Res. American Association for Cancer Research; 2010;70: 4569–4579. doi: 10.1158/0008-5472.CAN-09-2670 10.1158/0008-5472.CAN-09-2670 20460541

[44] Wang X, Tong X, Gao H, Yan X, Xu X, Sun S, et al Silencing HIWI suppresses the growth, invasion and migration of glioma cells. Int J Oncol. Spandidos Publications; 2014;45: 2385–2392. doi: 10.3892/ijo.2014.2673 25269862 10.3892/ijo.2014.2673

[45] Cao J, Xu G, Lan J, Huang Q, Tang Z, Tian L. High expression of piwi-like RNA-mediated gene silencing 1 is associated with poor prognosis via regulating transforming growth factor-β receptors and cyclin-dependent kinases in breast cancer. Mol Med Rep. Spandidos Publications; 2016;13: 2829–2835. doi: 10.3892/mmr.2016.4842 26847393 10.3892/mmr.2016.4842

[46] Dufourt J, Dennis C, Boivin A, Gueguen N, Théron E, Goriaux C, et al Spatio-temporal requirements for transposable element piRNA-mediated silencing during Drosophila oogenesis. Nucleic acids research. Oxford University Press; 2014;42: 2512–2524. doi: 10.1093/nar/gkt1184 24288375 10.1093/nar/gkt1184 PMC3936749

[47] Zhang Z, Wang J, Schultz N, Zhang F, Parhad SS, Tu S, et al The HP1 homolog rhino anchors a nuclear complex that suppresses piRNA precursor splicing. Cell. 2014;157: 1353–1363. doi: 10.1016/j.cell.2014.04.030 24906152 10.1016/j.cell.2014.04.030 PMC4167631

[48] Chen Y-CA, Stuwe E, Luo Y, Ninova M, Le Thomas A, Rozhavskaya E, et al Cutoff Suppresses RNA Polymerase II Termination to Ensure Expression of piRNA Precursors. Molecular cell. 2016;63: 97–109. doi: 10.1016/j.molcel.2016.05.010 27292797 10.1016/j.molcel.2016.05.010 PMC4980073

