# Supplementary material for: Cbp80 is needed for the expression of piRNA components and piRNAs
Source: PLoS One. 2017 Jul 26;12(7):e0181743. doi: 10.1371/journal.pone.0181743 (PMC5528831; doi:10.1371/journal.pone.0181743)
Supplement: S1 Fig — pUASP-Venus::Cbp80 tagged fly lines were crossed to different germline-specific GAL4 drivers. Ovaries from the resulting flies were dissected and DNA was stained with Hoechst. Venus signal is mostly nuclear. Scale bar: 25 μm. (PDF) [file pone.0181743.s001.pdf]

Supporting information S1

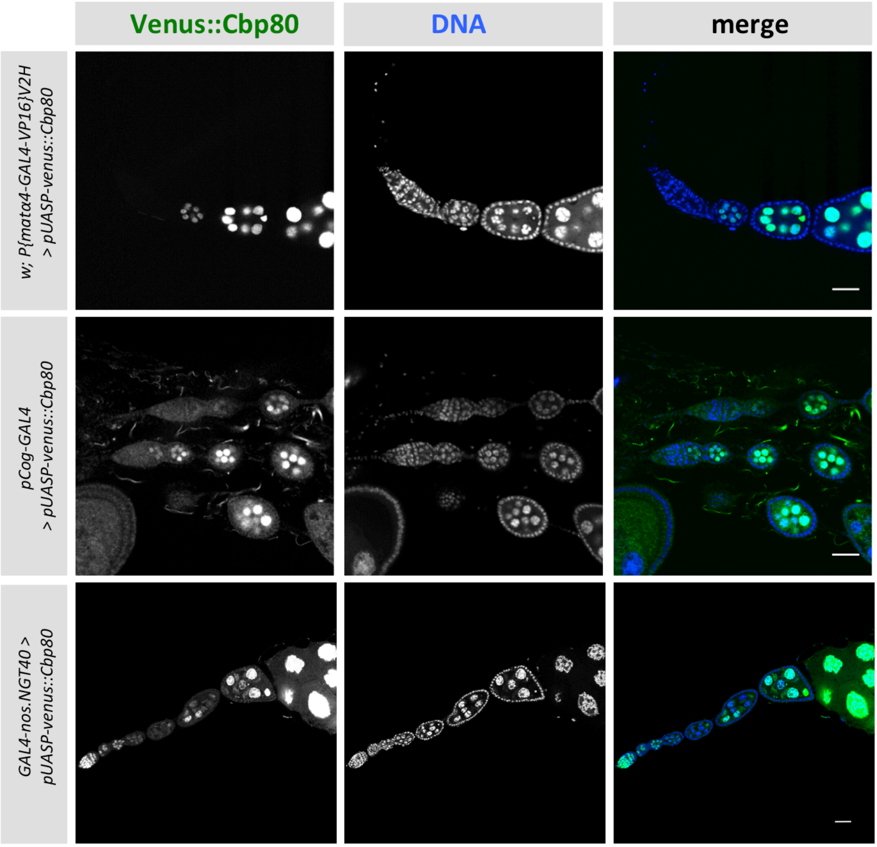

Expression pattern of germline-specific GAL4 drivers. *pUASP*-Venus::*Cbp80* tagged fly lines were crossed to different germline-specific GAL4 drivers. Ovaries from the resulting flies were dissected and DNA was stained with Hoechst. Venus signal is mostly nuclear. Scale bar: 25 µm.
