# Supplementary material for: Cbp80 is needed for the expression of piRNA components and piRNAs
Source: PLoS One. 2017 Jul 26;12(7):e0181743. doi: 10.1371/journal.pone.0181743 (PMC5528831; doi:10.1371/journal.pone.0181743)
Supplement: S2 Fig — Ovaries expressing specifically in the germline (pCog-Gal4 driver) shRNAs against Cbp80 or mCherry (as control) were used. Ovaries showing normal appearance upon Cbp80 knockdown (“n” phenotype; Fig 1), underdeveloped ovaries ("d" phenotype; Fig 1) and control ovaries were tested for Cbp80 levels by Western blotting. Tubulin was used as a loading control. 2 different amounts of each Cbp80 knockdown sample were loaded. (PDF) [file pone.0181743.s002.pdf]

Supporting information S2

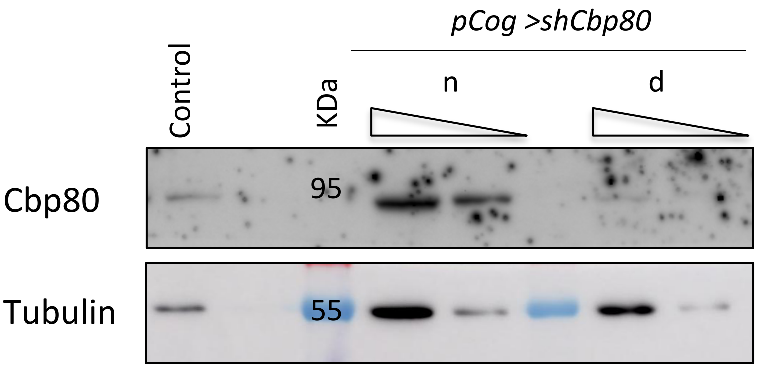

Cbp80 protein levels correlate with the severity of the different phenotypes observed upon *Cbp80* knockdown. Ovaries expressing specifically in the germline (pCog-Gal4 driver) shRNAs against *Cbp80* or *mCherry* (as control) were used. Ovaries showing normal appearance upon *Cbp80* knockdown (“n” phenotype; Fig. 1), underdeveloped ovaries ("d" phenotype; Fig. 1) and control ovaries were tested for Cbp80 levels by Western blotting. Tubulin was used as a loading control**.** 2 different amounts of each *Cbp80* knockdown sample were loaded.
