# Supplementary material for: Cbp80 is needed for the expression of piRNA components and piRNAs
Source: PLoS One. 2017 Jul 26;12(7):e0181743. doi: 10.1371/journal.pone.0181743 (PMC5528831; doi:10.1371/journal.pone.0181743)
Supplement: S3 Fig — Ovaries displaying the "d" phenotype upon Cbp80 knockdown were used in all experiments. (A-C) Fold increase in RNA levels of indicated TEs upon germline-specific RNAi-mediated knock down of Cbp80 (shRNA against Cbp80). The germline GAL4 driver alone was used as control. (A-B) Fold-changes in transposon RNA levels were normalized to rp49, Tub and BicD levels. Control ovaries expressed the shmCherry construct. (C) Levels of transposon transcripts relative to the control sample are shown. The same amount of total RNA was used as starting material. Error bars represent +/- SD of 2 control and 3 biological knock down replicates. (D-F) Fold increase in RNA levels of the same TEs upon germ line specific knock down of Cbp80 using dsRNA. (D-E) Fold changes relative to rp49, Tub and BicD. Control ovaries expressed a dsGFP RNAi construct. (F) Levels of transposon transcripts relative to the control sample are shown. The same amount of total RNA was used as starting material. Error bars represent +/- SD of 3 biological replicates. *p<0.05; **p<0.01; ***p<0.001. (PDF) [file pone.0181743.s003.pdf]

Supporting information S3

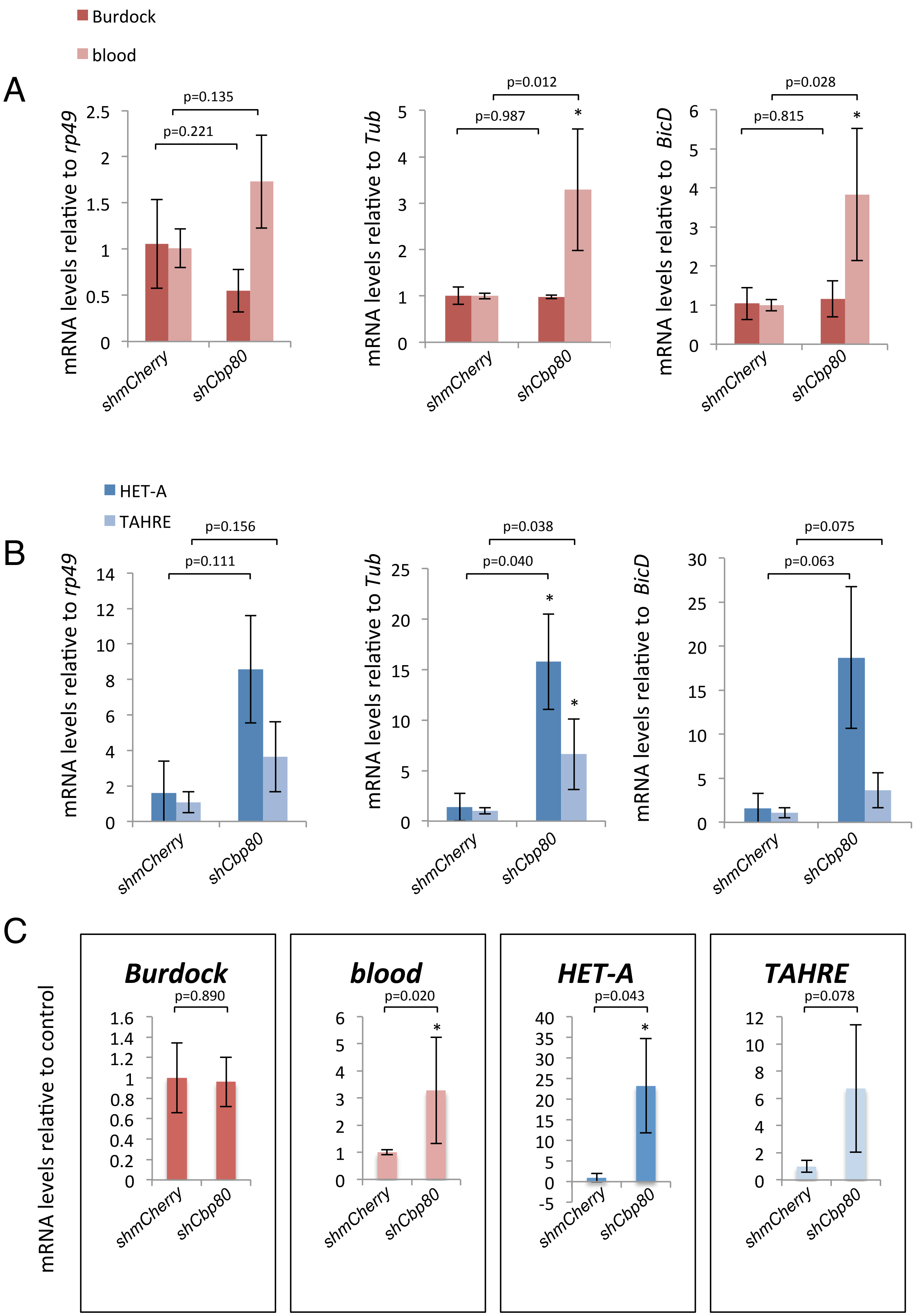

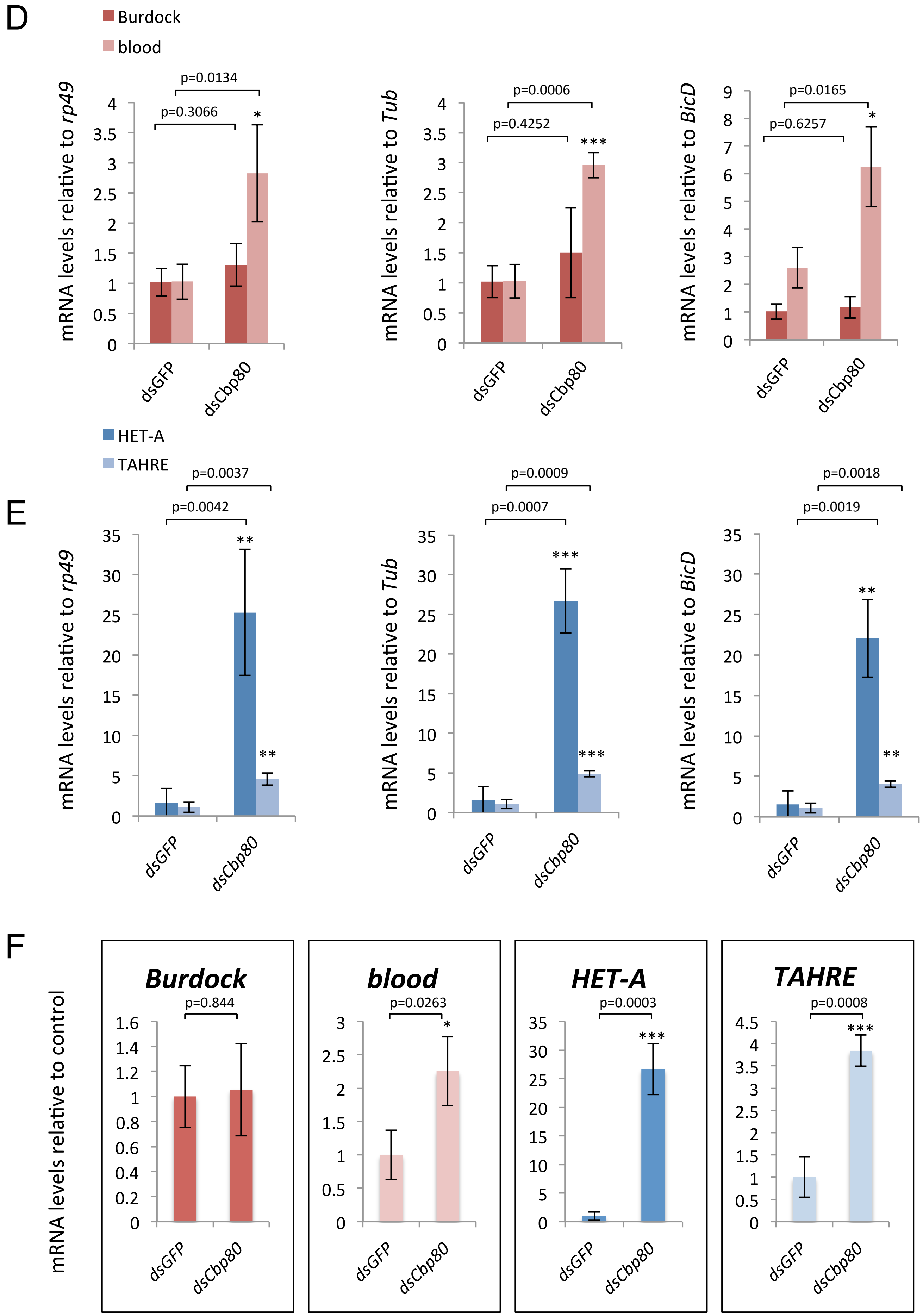
D
Burdock
blood
mRNA levels relative to rp49
mRNA levels relative to Tub
mRNA levels relative to BicD
p=0.0134
p=0.3066
p=0.0006
p=0.4252
p=0.0165
p=0.6257
dsGFP
dsCbp80
E
HET-A
TAHRE
p=0.0037
p=0.0042
p=0.0009
p=0.0007
p=0.0018
p=0.0019
F
mRNA levels relative to control
Burdock
blood
HET-A
TAHRE
p=0.844
p=0.0263
p=0.0003
p=0.0008

Upregulation of transposons (TEs) upon *Cbp80* knockdown.

Ovaries displaying the "d" phenotype upon *Cbp80* knockdown were used in all experiments. (A-C) Fold increase in RNA levels of indicated TEs upon germline-specific RNAi-mediated knock down of *Cbp80 (*shRNA against *Cbp80*). The germline GAL4 driver alone was used as control. (A-B) Fold-changes in transposon RNA levels were normalized to *rp49, Tub* and *BicD* levels. Control ovaries expressed the *shmCherry* construct. (C) Levels of transposon transcripts relative to the control sample are shown. The same amount of total RNA was used as starting material. Error bars represent $^{+}$/- SD of 2 control and 3 biological knock down replicates. (D-F) Fold increase in RNA levels of the same TEs upon germ line specific knock down of *Cbp80* using *dsRNA.* (D-E) Fold changes relative to *rp49, Tub* and *BicD.* Control ovaries expressed a *dsGFP* RNAi construct. (F) Levels of transposon transcripts relative to the control sample are shown. The same amount of total RNA was used as starting material. Error bars represent $^{+}$/- SD of 3 biological replicates. *p<0.05; **p<0.01; ***p<0.001.
