# Supplementary material for: Cbp80 is needed for the expression of piRNA components and piRNAs
Source: PLoS One. 2017 Jul 26;12(7):e0181743. doi: 10.1371/journal.pone.0181743 (PMC5528831; doi:10.1371/journal.pone.0181743)
Supplement: S4 Fig — Genotypes of ovaries analyzed are depicted on top of the figures. Histogram showing small RNAs (23–29 nucleotides long) mapping to the germline-specific uni-strand cluster 20A in flies expressing specifically in their germline shRNAs against Cbp80 or mCherry (as control). The germ line specific pCog-Gal4 driver was used for their expression. (PDF) [file pone.0181743.s004.pdf]

## Supporting information S4

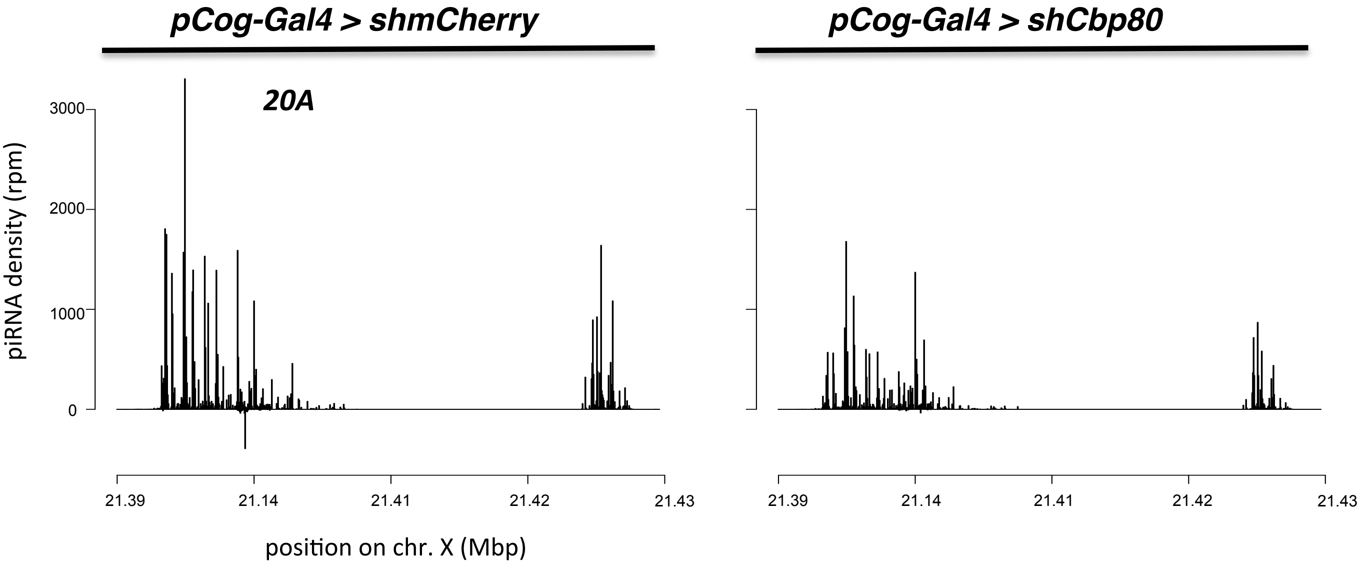


**Germline *Cbp80* is involved in producing piRNAs derived from the *20A* cluster.** Genotypes of ovaries analyzed are depicted on top of the figures. Histogram showing small RNAs (23-29 nucleotides long) mapping to the germline-specific uni-strand cluster *20A* in flies expressing specifically in their germline shRNAs against *Cbp80* or *mCherry* (as control). The germ line specific *pCog-Gal4* driver was used for their expression.
