# Supplementary material for: Cbp80 is needed for the expression of piRNA components and piRNAs
Source: PLoS One. 2017 Jul 26;12(7):e0181743. doi: 10.1371/journal.pone.0181743 (PMC5528831; doi:10.1371/journal.pone.0181743)
Supplement: S5 Fig — Cbp80 signal (green) is primarily seen inside the nucleus in Drosophila nurse cells. Nuclear compartments are delineated by the nuclear envelope protein Lamin (red). Single nurse cell nuclei are shown and the DNA is stained in blue. Scale bar: 10 μm. (PDF) [file pone.0181743.s005.pdf]

Supporting information S5

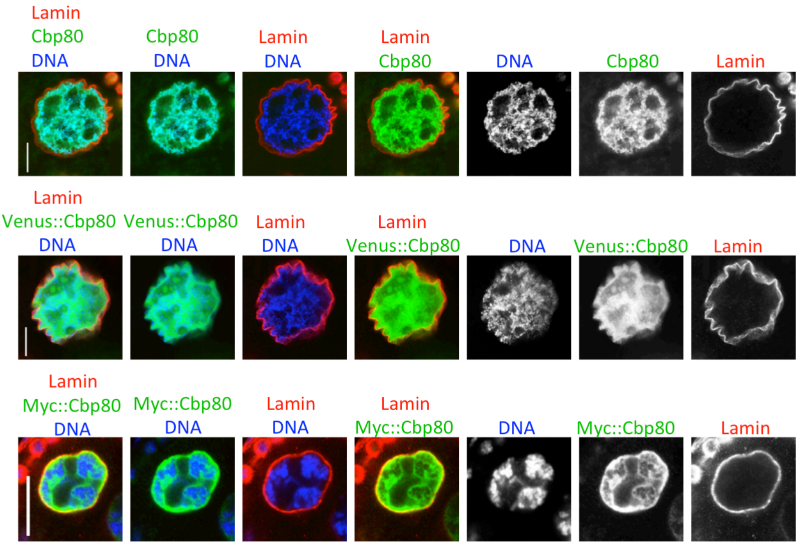

Tagged and untagged Cbp80 show similar subcellular accumulation patterns. Cbp80 signal (green) is primarily seen inside the nucleus in *Drosophila* nurse cells. Nuclear compartments are delineated by the nuclear envelope protein Lamin (red). Single nurse cell nuclei are shown and the DNA is stained in blue. Scale bar: 10 µm.
