# Supplementary material for: Cbp80 is needed for the expression of piRNA components and piRNAs
Source: PLoS One. 2017 Jul 26;12(7):e0181743. doi: 10.1371/journal.pone.0181743 (PMC5528831; doi:10.1371/journal.pone.0181743)
Supplement: S6 Fig — Interaction test of Cbp80 either in the DNA binding domain (BD, upper) or in the activator domain (AD, lower) vector with Piwi. Cbp20 and empty vectors were used as positive and negative controls, respectively. No interaction between Cbp80 and Piwi was detected in either case. (PDF) [file pone.0181743.s006.pdf]

Supporting information S6

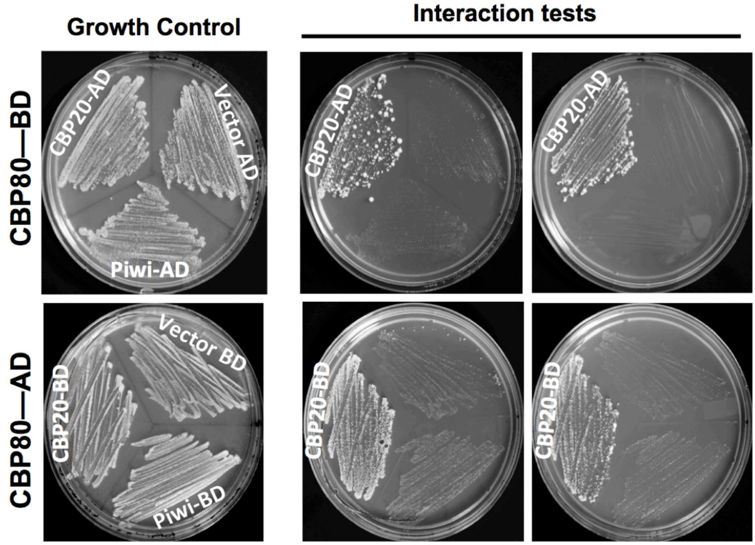

Piwi does not interact directly with Cbp80 in the yeast two-hybrid system. Interaction test of Cbp80 either in the DNA binding domain (*BD, upper*) or in the activator domain (*AD*, lower) vector with Piwi. Cbp20 and empty vectors were used as positive and negative controls, respectively. No interaction between Cbp80 and Piwi was detected in either case.
