# Supplementary material for: Cbp80 is needed for the expression of piRNA components and piRNAs
Source: PLoS One. 2017 Jul 26;12(7):e0181743. doi: 10.1371/journal.pone.0181743 (PMC5528831; doi:10.1371/journal.pone.0181743)
Supplement: S7 Fig — (A-B) Ovaries expressing specifically in the germline (pCog-Gal4 driver) shRNAs against Cbp80 or mCherry (as control). For the Cbp80 knockdown, only partially developed ovaries were collected. Ovarioles were stained at the same time for Lamin (blue), Cbp80 (green) and the piRNA precursor transcripts from clusters 42AB (red) and 20A (green). Left pictures show confocal images of nurse cell nuclei stained for Lamin and Cbp80. Right pictures show the signals for Lamin and the transcripts. Upper panels show an example of a control egg chamber with a clear nuclear Cbp80 signal. Lower panels show a Cbp80 knockdown example with strongly reduced nuclear Cbp80 staining. Levels and localization of the 42AB and 20A cluster transcripts show no clear change upon Cbp80 knockdown. (B) Anti-Lamin staining allowed us to classify perinuclear dots from the 42AB and 20A transcripts as residing in the ‘nuage’ region (if they were within approx. 1μm of the Lamin signal) or inside the nucleus. Dots overlapping with the Lamin signal were not counted. The percentage of transcripts in the ‘nuage’ (relative to transcripts in the ‘nuage’ and the nucleus) was ploted for control and Cbp80 knockdown. No significant differences were observed between them. (C) Ovaries expressing specifically in the germline (MTD-Gal4 driver) shRNAs against Rhi or white (as control) were used to test the specificity of the 42AB probe. Ovarioles were stained at the same time for Lamin, Rhi and the 42AB and 20A piRNA precursor transcripts. Left pictures show confocal images of nurse cell nuclei stained for Lamin (blue) and Rhi (green). Right pictures show the signals for Lamin (blue), 42AB transcripts (red) and 20A transcripts (green). The signal for the 42AB probe was lost upon Rhi knockdown, which affects transcription from this cluster, confirming the specificity of the probe and the in situ protocol used. Expression of the 20A cluster is not affected (as expected). (PDF) [file pone.0181743.s007.pdf]

Supporting information S7

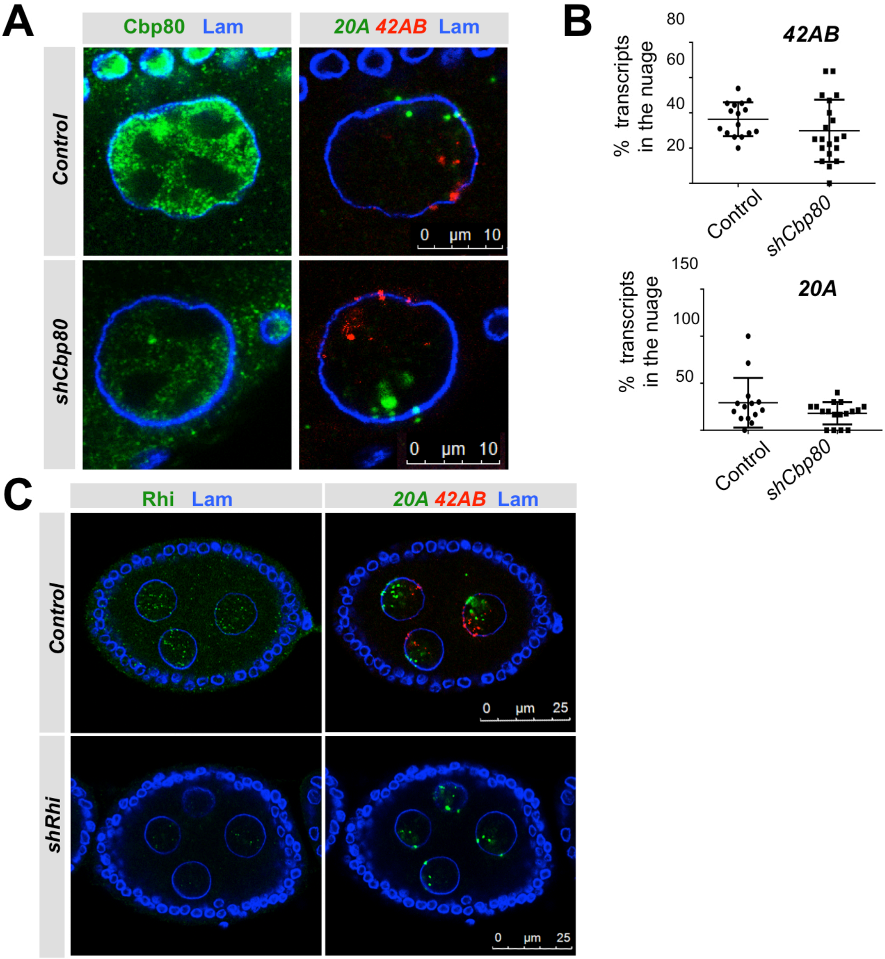

Export of piRNA precursors is not significantly affected by *Cbp80* knockdown. **(A-B)** Ovaries expressing specifically in the germline (pCog-Gal4 driver) shRNAs against *Cbp80* or *mCherry* (as control). For the *Cbp80* knockdown, only partially developed ovaries were collected. Ovarioles were stained at the same time for Lamin (blue), Cbp80 (green) and the piRNA precursor transcripts from clusters *42AB* (red) and *20A* (green). Left pictures show confocal images of nurse cell nuclei stained for Lamin and Cbp80. Right pictures show the signals for Lamin and the transcripts. Upper panels show an example of a control egg chamber with a clear nuclear Cbp80 signal. Lower panels show a *Cbp80* knockdown example with strongly reduced nuclear Cbp80 staining. Levels and localization of the *42AB* and *20A* cluster transcripts show no clear change upon *Cbp80* knockdown. **(B)** Anti-Lamin staining allowed us to classify perinuclear dots from the *42AB* and *20A* transcripts as residing in the 'nuage' region (if they were within approx. 1µm of the Lamin signal) or inside the nucleus. Dots overlapping with the Lamin signal were not counted. The percentage of transcripts in the 'nuage' (relative to transcripts in the 'nuage' and the nucleus) was ploted for control and *Cbp80* knockdown. No significant differences were observed between them. **(C)** Ovaries expressing specifically in the germline (MTD-Gal4 driver) shRNAs against *Rhi* or *white* (as control) were used to test the specificity of the *42AB* probe. Ovarioles were stained at the same time for Lamin, Rhi and the *42AB* and *20A* piRNA precursor transcripts. Left pictures show confocal images of nurse cell nuclei stained for Lamin (blue) and Rhi (green). Right pictures show the signals for Lamin (blue), *42AB* transcripts (red) and *20A* transcripts (green). The signal for the *42AB* probe was lost upon Rhi knockdown, which affects transcription from this cluster, confirming the specificity of the probe and the *in situ* protocol used. Expression of the *20A* cluster is not affected (as expected).
