# Supplementary material for: Cbp80 is needed for the expression of piRNA components and piRNAs
Source: PLoS One. 2017 Jul 26;12(7):e0181743. doi: 10.1371/journal.pone.0181743 (PMC5528831; doi:10.1371/journal.pone.0181743)
Supplement: S8 Fig — Ovaries expressed in their germ line shRNAs against Cbp80 (trip line) and shRNAs against mCherry (as control), respectively, under the pCog-Gal4 driver. Control flies expressed also a Jupiter-mCherry fusion gene in a jupiter+ background. Levels of precursor transcripts for two regions of the 42AB cluster, regions A and 1–32, and the flam locus were measured by qRT-PCR. Fold expression levels relative to the expression of rp49 are shown for each sample. Error bars represent +/-SD of 2 control and 3 biological knock down replicates. While there is a high variability between the different biological samples (probably due to the phenotypic differences between the knock down samples and the size-matched wild-type stages), no reduction in the expression of piRNA precursors was observed upon Cbp80 knockdown. (PDF) [file pone.0181743.s008.pdf]

Supporting information S8

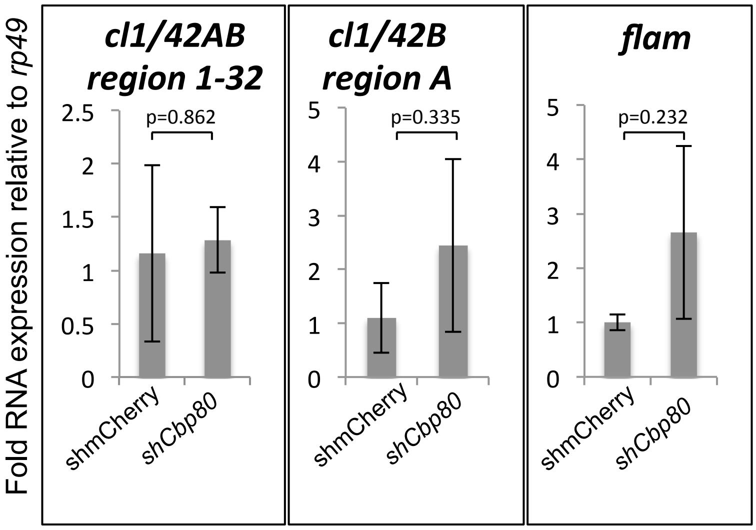

Levels of *42AB* precursor transcripts are not reduced upon *Cbp80* knockdown.
Ovaries expressed in their germ line shRNAs against *Cbp80 (trip line*) and shRNAs against *mCherry* (as control*),* respectively, under the pCog-Gal4 driver. Control flies expressed also a *Jupiter-mCherry* fusion gene in a *jupiter*$^{+}$ background. Levels of precursor transcripts for two regions of the *42AB* cluster, regions *A* and *1-32,* and the *flam* locus were measured by qRT-PCR. Fold expression levels relative to the expression of *rp49* are shown for each sample. Error bars represent $^{+}$/-SD of 2 control and 3 biological knock down replicates. While there is a high variability between the different biological samples (probably due to the phenotypic differences between the knock down samples and the size-matched wild-type stages), no reduction in the expression of piRNA precursors was observed upon *Cbp80* knockdown.
