# Supplementary material for: Cbp80 is needed for the expression of piRNA components and piRNAs
Source: PLoS One. 2017 Jul 26;12(7):e0181743. doi: 10.1371/journal.pone.0181743 (PMC5528831; doi:10.1371/journal.pone.0181743)
Supplement: S9 Fig — (A) Ovaries expressing specifically in the germline (pCog-Gal4 driver) shRNAs against Cbp80 (shCbp80) or mCherry (shmCherry; as control) were used. Control flies expressed also a Jupiter-mCherry fusion protein. (B) Egg chambers expressing a dsRNA against Cbp80 (dsCbp80) under the control of the GAL4-nos.NGT40 driver combined with the UAS-Dcr2. Control flies expressed a dsRNA against GFP (dsGFP). (A-B) Ovaries showing the "d" phenotype (Fig 1) upon Cbp80 knockdown were used. mRNA levels of piRNA pathway factors were tested by qRT-PCR. The expression of the piwi component mRNAs was normalized relative to the expression of control genes (Tub, BicD and rp49). Error bars represent +/- SD of 2 control (in B) and 3 control samples (in A) and 3 biological knock down replicates. *p<0.05; **p<0.01; ***p<0.001. (PDF) [file pone.0181743.s009.pdf]

Supporting information S9

A

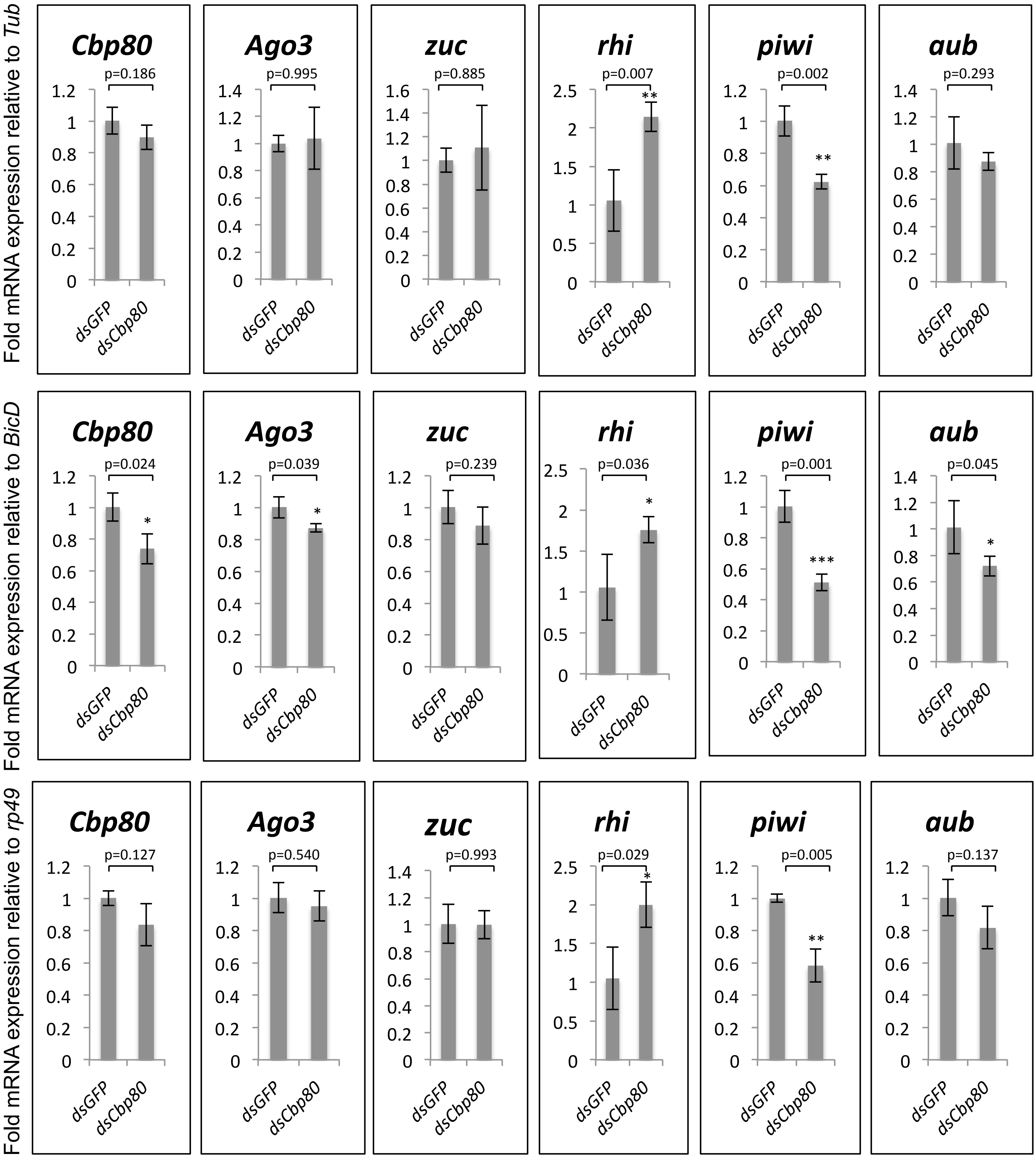

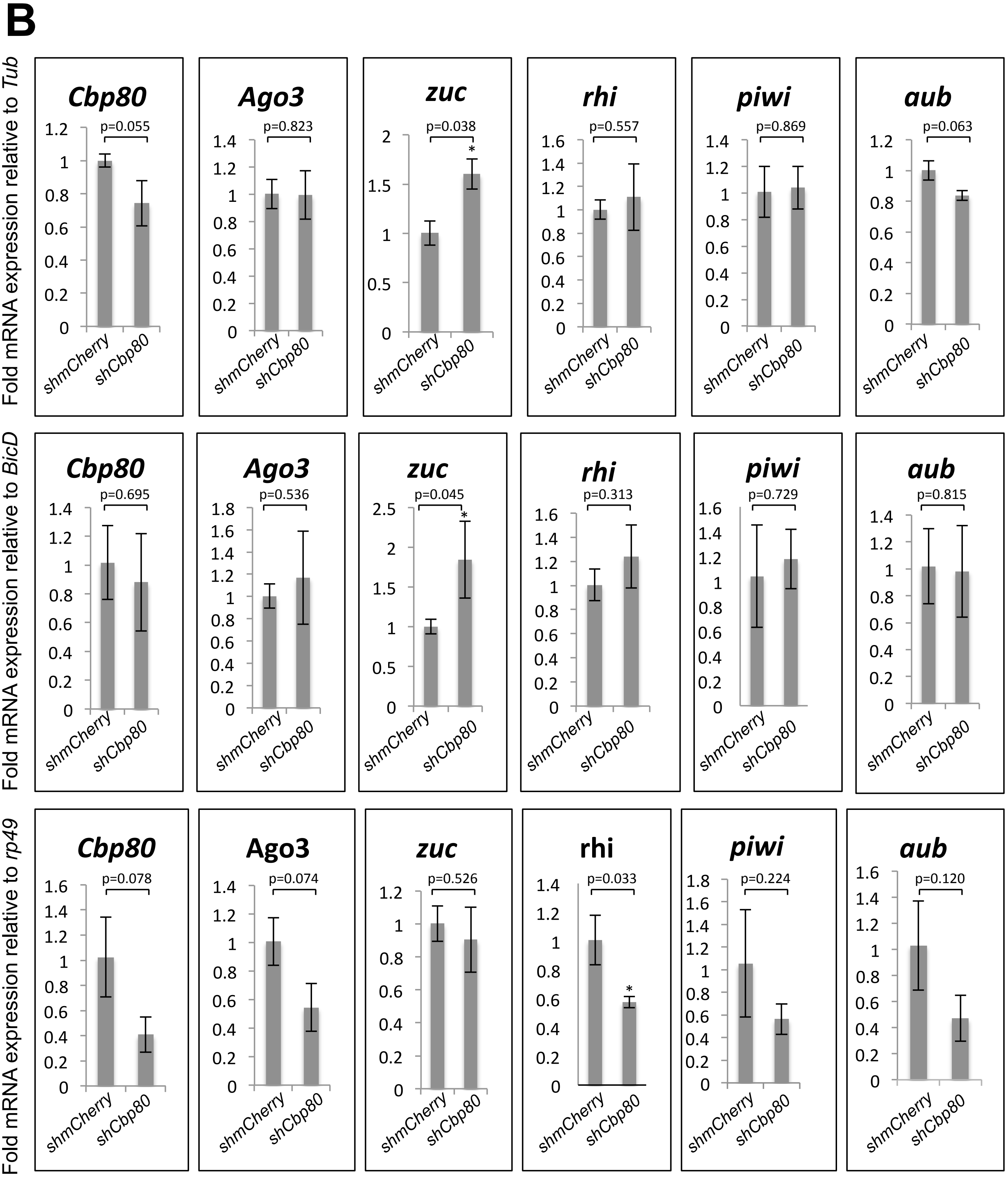
B
Fold mRNA expression relative to Tub
Fold mRNA expression relative to BicD
Fold mRNA expression relative to rp49
Cbp80
Ago3
zuc
rhi
piwi
aub
p=0.055
p=0.823
p=0.038
p=0.557
p=0.869
p=0.063
p=0.695
p=0.536
p=0.045
p=0.313
p=0.729
p=0.815
p=0.078
p=0.074
p=0.526
p=0.033
p=0.224
p=0.120
shmCherry
shCbp80

mRNAs coding for piRNA pathway components display different sensitivities to *Cbp80* knockdown. (A) Ovaries expressing specifically in the germline (pCog-Gal4 driver) shRNAs against *Cbp80 (shCbp80)* or *mCherry* (*shmCherry*; as control) were used. Control flies expressed also a Jupiter-mCherry fusion protein. **(B)** Egg chambers expressing a *dsRNA* against *Cbp80 (dsCbp80*) under the control of the GAL4-nos.NGT40 driver combined with the *UAS-Dcr2***.** Control flies expressed a *dsRNA* against *GFP* (*dsGFP*). **(A-B)** Ovaries showing the "d" phenotype (Fig. 1) upon *Cbp80* knockdown were used. mRNA levels of piRNA pathway factors were tested by qRT-PCR. The expression of the piwi component mRNAs was normalized relative to the expression of control genes (*Tub*, *BicD* and *rp49*). Error bars represent $^{+}$/- SD of 2 control (in B) and 3 control samples (in A) and 3 biological knock down replicates. *p<0.05; **p<0.01; ***p<0.001.
